# Pathways to School Reentry for Children and Young People with a Medical or Mental Health Condition: An International Delphi Study

**DOI:** 10.5334/cie.159

**Published:** 2025-03-05

**Authors:** Michele Capurso, Valentina Moracci, Simone Borsci

**Affiliations:** 1 Università degli Studi di Perugia, IT; 2 Independent researcher, IT; 3 Department Learning, Data analytics and Technology – CoDe Human Factors group, Faculty BMS, University of Twente, NL; 4 London NIHR Healthtech Research Centre in In Vitro Diagnostics, Imperial College of London, UK

**Keywords:** pediatric hospitalization, school reentry, student, pupil, learner, medical or mental health condition, chronic illness, education

## Abstract

Prolonged school absences among children and young people hospitalized due to medical or mental health conditions can significantly disrupt their social and academic development. This study addresses the critical process of reintegrating these learners into their school environments and develops a comprehensive, consensus-based model to facilitate successful school reentry. Utilizing the Delphi method, the research collected insights from 56 experts across 18 countries, representing education, healthcare, and academia, over three rounds of consultation between 2022 and 2024. The findings identify key temporal phases and actions crucial for planning and executing reentry strategies. The resulting models (one for physical health conditions and another for mental health conditions) offer structured guidance, emphasizing multidisciplinary collaboration, context-specific adaptations, and the active participation of the students in the process. The study underscores the need for tailored interventions that address the unique challenges faced by each learner, emphasizing the importance of integrating educational, healthcare, and social support systems with the local culture and values of children and their families to foster resilience and successful reintegration.

According to 2019 United States (U.S.) data, pediatric inpatient discharges (excluding birth hospitalization) totaled 1,671,515 individuals (Steiner et al., 2023). Considering a population of approximately 70,000,000 for the same age group (U.S. Census Bureau, 2019), around 2.38% of the pediatric population were hospitalized in 2019. Therefore, each teacher is likely to encounter the hospitalization of one or more of their learners reasonably often during their career. While the average hospital stay is relatively brief (around 4.1 days, Weiss, Liang, & Martin, 2022), children and young people with severe illnesses may be hospitalized for extended periods and often need to stay home for longer before returning to school.

Hospitalization and related school absences can significantly disrupt social and academic aspects of school life as well as children and young people’s development. Prolonged absences from school may result in interrupted learning, creating substantial gaps in a learner’s academic knowledge and skills, making it challenging to keep pace with their peers (Wikel & Markelz, 2023). The lack of consistent classroom attendance also affects essential social interactions. Children and young people with chronic illnesses often miss opportunities to develop and practice social skills, leading to feelings of isolation and difficulties maintaining friendships (Harris, 2009). These social disruptions can exacerbate emotional distress, compounding the challenges of reentry into the school environment. Furthermore, the physical and emotional toll of their medical or physical conditions can lead to fatigue, anxiety, and reduced participation in school activities, further alienating these students from their classmates and the wider school community (Faulkner, Trenchard, Taylor, & Murray, 2021; Shiu, 2001).

Psychiatric hospitalizations for mental health diagnoses in the U.S. in 2019 reached 201,932 (Arakelyan et al., 2023), accounting for approximately 12% of all pediatric hospitalizations in the same year. Like the disruptions caused by physical illnesses, hospitalization for mental health conditions affects the social and academic lives of children and young people, while introducing unique challenges (Tougas, Houle, Leduc, Frenette-Bergeron, & Marcil, 2023). In mental health, the transition from school to hospital is often abrupt. It can be overwhelming for the child, their families, the teachers, and schoolmates, due to the intense nature of psychiatric care and the stigma often associated with psychiatric issues (Clemens, Welfare, & Williams, 2011). Furthermore, the requirement for continuous mental health care, including therapy sessions and medication management, can further disrupt the school routine and intensify the difficulties associated with reintegration (Tougas, Rassy, Frenette-Bergeron, & Marcil, 2019). This example highlights the critical importance of enhancing collaboration between acute psychiatric hospitals and schools because when a student’s psychiatric needs are not adequately prioritized following discharge, the likelihood of successful school reintegration is significantly reduced, often leading to rehospitalization (Tougas et al., 2023).

Previously, normal schooling and education were frequently interrupted due to hospitalizations (Lee, 1975), with education often being treated as a secondary concern in the face of health threats. However, during the 1970s, many countries progressively embraced a biopsychosocial model of health care (Engel, 1977), which emphasized providing educational services and developmental support as being vital components of health and recovery for young patients within children’s hospitals and beyond (Cahners, 1979). School is now widely recognized as a standard and integral component of many healthcare services, encompassing physical and mental health (Castro Ibáñez, 2023). Ideal school programs emphasize school attendance as a central goal to help reintegrate the child into the academic environment and to represent a return to normalcy (Shaw & McCabe, 2008). Direct narratives of hospitalized children show how the significance of school extends beyond cultural and cognitive development; school plays a crucial role in fostering interpersonal and intrapersonal skills such as emotional regulation and relationship management (Capurso, di Castelbianco, & Di Renzo, 2021). These skills are important for children and adolescents with chronic illnesses, as they contribute significantly to their development and are major determinants of success in education and future employment (Kushner & Rengasamy, 2024). While it is well established that school is vital to the intellectual, social, and emotional well-being of children, research indicates that those with chronic illnesses often experience poorer psychological adjustment and are more prone to emotional and behavioral issues than their healthy peers (Layte & McCrory, 2013).

While education of children and young people with physical or mental health conditions is vital, it is also becoming progressively more complex (Galligan & Hogan, 2021). Plsek and Greenhalgh (2001) addresses the increasing complexity in healthcare and presents key systemic concepts pertinent to educational practices within a healthcare system at organizational and pedagogical levels. The school operates as one system (Hopkins, Stringfield, Harris, Stoll, & Mackay, 2014), and when it works with children and young people who are currently or have previously been hospitalized, it interacts with other complex adaptive systems composed of multiple interacting agents such as parents, siblings, schoolmates, healthcare providers, and social services (Tougas et al., 2019). Each of these systems possess a certain degree of freedom, allowing them to act in unpredictable ways. According to Plsek and Greenhalgh (2001), such interactions are characterized by fuzzy boundaries, implicit and explicit rules driving the actions of different professionals, adaptability, embeddedness within other systems, inherent tensions and paradoxes, emergent behaviors, non-linearity, and a degree of unpredictability.

Organization at a different system level is essential to respond to this complexity (Sameroff, 2010). Nursing research provides further insights into effective and respectful ways of managing relationships with children and young people with medical or mental health needs and their families. These include respecting the routines of parents and children, cultivating trust and empathy, engaging in meaningful negotiation and support, advocating and facilitating informed choices, and fostering knowledge exchange and coordination. Other findings emerge from review studies within education. For instance, regarding learners with physical or mental health needs, Clemens et al. (2011) emphasizes the importance of consistent communication between stakeholders and the development of a specific, individualized reentry plan before discharge, which should be coupled with a plan that facilitates continuity of care, especially for learners with mental health needs. Vanclooster, Benoot, Bilsen, Peremans, and Jansen (2018) highlights the importance of a school liaison figure to enhance ongoing knowledge, education, and support related to the learner’s physical condition. Similarly, Canter and Roberts (2012) underscores the value of programs aimed at increasing specific knowledge of the learner’s situation and fostering positive attitudinal changes in schools.

Other key concepts from the International Classification of Functioning, Disability, and Health (ICF) are essential in planning school reentry. In fact, ICF provides a framework to understand how barriers and facilitators affect an individual’s functioning within a biopsychosocial context (World Health Organization, 2007). Facilitators include positive attitudes, supportive policies, accessible environments, and tools that help individuals overcome health-related limitations and enhance participation in society. In education, this might involve family support, motivation, and tailored programs to help children and young people with health needs engage effectively (de Rooij, van de Port, van der Heijden, Meijer, & Visser-Meily, 2021). Conversely, barriers – such as inaccessible infrastructure, discriminatory attitudes, or inadequate policies – hinder full participation and exacerbate the challenges faced by students and their families (Schneidert, Hurst, Miller, & Üstün, 2003).

Effective communication and well-coordinated reentry plans involving the pupils and students, educators, medical and mental health professionals, and families are crucial to overcoming barriers and facilitating a smoother transition back to school (Fotheringham, Karabon, Wunderlich-Barillas, Traynor, & Gowans, 2021; Midura, Fodstad, White, Turner, & Menner, 2023). Given that school reentry programs have been implemented for several decades worldwide, it is worth assessing the common principles drawn from these experiences and an

## Definitions of terms

This article is the result of an international study involving dozens of scholars worldwide. To ensure consistency throughout our research, the following terms have been used and/or suggested during our Delphi rounds and applied in the present paper.

### Learner

An inclusive designation for individuals of any gender and age engaged in educational activities at primary and secondary school levels. This term underscores the dynamic and continuous nature of learning, emphasizing the active involvement, autonomy, and developmental growth of individuals in the learning process. By choosing *learner* over more traditional terms such as *pupil* or *student*, the focus shifts toward the holistic experience, unique needs, and agency of each individual, thereby aligning with inclusive and personalized educational approaches (Lerner, Liben, & Mueller, 2015).

### Children and young people

Encompasses the broader aspects of life and development of individuals, extending beyond the educational sphere. This term acknowledges the multifaceted nature of their experiences and growth, recognizing that their development is influenced by a wide array of factors beyond formal education.

### School reentry

The structured educational process that facilitates and supports learners’ return to—and successful reintegration into—their original school environment, after an extended absence resulting from mental or physical health conditions, prolonged hospitalization, or other disruptions to regular school attendance (adapted from Schilling & Getch, 2018, p. 1028).

### Mainstream school

The school that the learner typically attends.

### Home schooling

The situation where a learner is taught at home due to illness, preventing attendance at a mainstream school. In this case, they may receive home visits from a teacher and/or engage in online learning.

### Time phases

The distinct key moments that characterize the process of a school reentry journey. These phases typically outline the critical stages or periods during which significant actions, decisions, or changes occur, marking the progression through the reentry process. They are represented on the generated models as vertical columns.

### Actions

The specific key areas of action that must be established and implemented when planning and executing a process to facilitate the school reentry of an absent learner. These actions are represented in our models as different horizontal rows developing progressively throughout the various time phases.

## Study Objective

This research had three primary objectives: 1) identify the critical time phases necessary for developing an effective school reentry program for learners with medical and/or mental health needs, 2) for each distinct time phase and with consideration of potential barriers and facilitators, identify key actions, compile specific activities, best practices, illustrative case materials, and provide guidelines to develop culturally adapted strategies that facilitate the school reentry process, and 3) measure the overall expert agreement regarding the suggested time phases and corresponding actions.

## Methods

### Design

This study employed a qualitative descriptive design, a methodology frequently utilized in healthcare research (Doyle, McCabe, Keogh, Brady, & McCann, 2020). This approach offers clear and direct descriptions of the experiences, perceptions, and practices of a diverse group of professionals from various healthcare and educational settings worldwide. The qualitative descriptive design was deemed most appropriate for our study as it acknowledges the contextual and cultural nature of the interventions put in place to facilitate school reentry and the fact that the experiences of the participants would vary based on local healthcare and education systems (Bradshaw, Atkinson, & Doody, 2017).

We employed the Delphi method to gather the information necessary to answer our research questions. This method utilizes a structured process of questionnaires and controlled feedback to leverage the collective intelligence of a panel of experts, enhancing knowledge on complex issues or forecasts (Linstone & Turoff, 2002). While a traditional Delphi study aimed to gain consensus from a group of experts on a specific problem (Dalkey & Helmer, 1963), over time, the Delphi technique has evolved into several varieties, each with its own specific focus and approach (Keeney, Hasson, & McKenna, 2011; Linstone & Turoff, 2002). For instance, non-consensus variants like the Policy Delphi are used to support decision-making or explore diverse perspectives on complex issues (Crisp, Pelletier, Duffield, Adams, & Nagy, 1997).

### Delphi steering workgroup

A small Delphi workgroup comprising three people was assembled to conduct this study. MC, who conceptualized this work, is an associate professor in developmental and educational psychology with previous experience as a hospital teacher. He is also active in academic research in hospital schools and the education of children with medical needs. He was part of an EU-funded project on these subjects and served as a member of the board of the Hospital Organization of Pedagogues in Europe for eight years. SB is currently working as an associate professor of Human Factors and Cognitive Ergonomics and holds the position of Honorary Senior Fellow of Human Factors for Health Technology at Imperial College London. SB’s research focuses on evaluating the quality of technology, processes, and artifacts to enhance interaction, communication, and experiences, particularly in health and disability contexts. SB has also authored other Delphi-based articles. At the time of this research, VM had recently graduated from an undergraduate psychology program in Italy and completed a postgraduate specialized program in scientific research. VM participated in this work as part of her thesis credits for her master’s degree in clinical and health psychology.

### The four rounds of the present study

**Figure 1:**
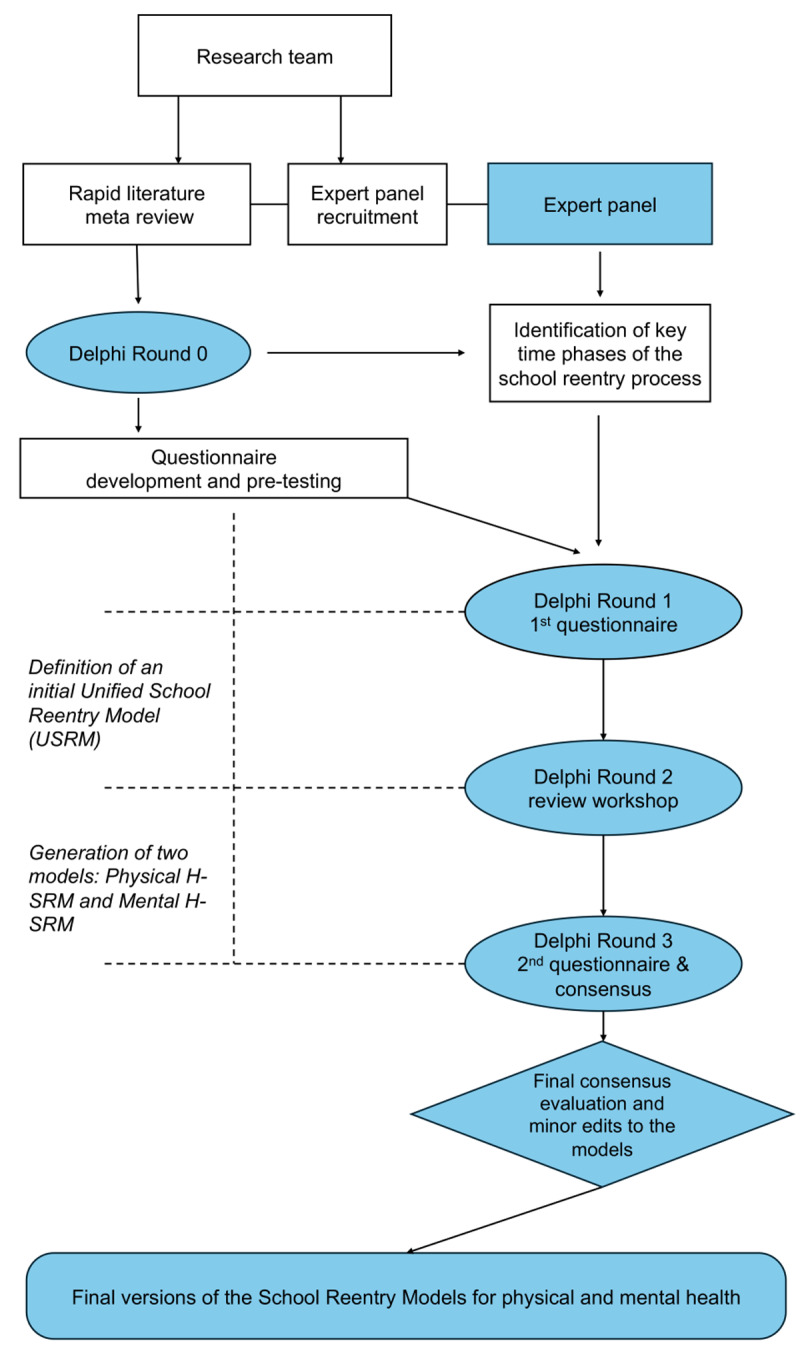
Flow chart depicting the mixed Delphi method study protocol. A steering workgroup completed the initial rapid meta review, drafted the questions for the different Delphi rounds, and organized the results.

This study utilized a Mixed Delphi Method (Bloor, Sampson, Baker, & Dahlgren, 2015), which included an initial preparation (Round 0) and three further Delphi rounds, organized as follows (See Figure 1).

#### Round 0

A literature review is often used as a starting point to frame the theoretical background and assist in formulating initial statements for the subsequent Delphi rounds (Belton, MacDonald, Wright, & Hamlin, 2019). In our study, a Rapid Meta Review (Wong, Greenhalgh, Westhorp, Buckingham, & Pawson, 2013) was performed to identify key time phases in the school reentry process for learners with medical and mental health conditions. A search was conducted of the following databases: Eric, Scopus, and Pubmed. Review articles were searched using the following keywords; academic achievement, adolescent disease, childcare, childhood cancer, childhood disease, chronic disease, education, mental health care, program, quality of life, reentry, reintegration, return, review, school, and student.

#### Round 1

A Policy Delphi (Crisp et al., 1997) was conducted online from 15^th^ November 2022 to 15^th^ January 2023, using the Qualtrics© platform to identify and evaluate factors that facilitate the return to school for learners with medical or mental health conditions after an extended absence. Based on their expertise, experts were first asked to specify the type of condition—mental or physical—they would focus on when discussing school reentry. They were then asked to (i) express their overall agreement with the proposed time phases using a 7-point Likert scale (ranging from 1 = strongly disagree to 7 = strongly agree); and (ii) suggest specific actions for each time phase that would facilitate school reentry. The questionnaire addressed pre-reentry actions during initial hospital admission, hospital stay, discharge, home education, and actions during the reentry and post-reentry phases. Respondents were also invited to provide feedback on the clarity of the questions and suggest topics for future rounds, with a focus on best practices, challenges, and factors aiding the transition from hospital to school. This round generated an initial Unified School Reentry Model (USRM; see the results section) that was subsequently used as input for Round 2.

#### Round 2

A Real-Time Delphi review workshop (Gordon & Pease, 2006) was held during a face-to-face meeting organized on the pre-congress days of the 12th HOPE Congress in Milan, Italy, from May 8–12, 2023. This round discussed and provided feedback on the USRM, as organized and presented by the Delphi team. After an initial discussion, participants proposed splitting the USRM into two versions: one for learners with mental health conditions and another for those with physical health conditions. Consequently, although the input questionnaire remained the same for all participants, to better address specific actions of the USRM, participants were further divided into subgroups where they engaged in a structured discussion focused on the questionnaire within their area of expertise. The questionnaire was centered around a table pertinent to the different time phases of the school reentry process identified during Round 0. Each table contained a description of the actions gathered during Round 1. Each subgroup was assigned a single action. Participants were instructed to familiarize themselves with the outlined actions. Following this, they were prompted to reflect on a set of questions that aimed to elicit additional suggestions for (i) content or suggestions that should be added to the model, (ii) content or suggestions that were deemed inappropriate or unsuitable, and (iii) practical ideas and tools to improve the practical application of the model. After the reflection period, participants engaged in a group discussion for ten minutes, during which they shared their insights and deliberated on their comments as a group. The group then collaboratively summarized their answers, documenting them on the subsequent pages of the paper containing the table they were discussing. To facilitate comprehensive input and diverse perspectives, space was left for additional comments from other group members. After the discussion period, as directed by the meeting chair, each group passed their results for their specific action to the next group, ensuring a continuous exchange of ideas and feedback across all participating groups. This process was repeated three times to harness the collective expertise and foster a thorough exploration of strategies to support students’ reentry. As presented in the results section, this round generated two distinct models, the Physical Health – School Reentry Model (H-SRM) and the Mental H-SRM, which served as the bases for Round 3.

#### Round 3

A final classical Consensus Delphi was conducted online (Dalkey & Helmer, 1963) from 15^th^ January 2024 to 1^st^ March 2024 to assess the level of agreement regarding the time phases and actions identified and described in the preceding rounds. This round aimed to gauge consensus on whether each one of the two models generated through Round 2 accurately depicted the necessary actions and stages to support learners. Respondents were instructed to rate their agreement on a 7-point Likert scale for each group of actions within the identified action of the reentry process, regardless of local implementation constraints. They were encouraged to adopt general and international perspectives rather than consider only their specific work environments and could also add verbal comments for each one of the identified actions. Furthermore, participants addressed two contentious issues that arose from earlier rounds; the preference for quantitative versus qualitative approaches in evaluating learners’ readiness to return to their school of belonging, and the appropriateness of using apps and games that framed their illness or condition as a battle to be fought. The final versions of the Physical H-SRM and Mental H-SRM, along with the experts’ agreement scores, are presented in detail in the results section.

### Ethics

The Delphi participants were informed that by participating in the research they authorized the use of their answers in the survey. All contact information was treated with confidentiality and all answers were anonymized. The study was approved by the Ethics Committee of the BMS faculty of the University of Twente (NL) protocol number 220920.

### Data Analysis

Interquartile range (IRQ), mean, and standard deviation were used to examine agreement in Rounds 1 and 3 (von der Gracht, 2012). Specifically, following suggestions from health care studies, agreement was established when IRQ ≤ 2 and the mean score was ≥ 5.5 (Polisena et al., 2018).

Concerning the qualitative data analysis, we employed a thematic synthesis method for Round 0 (Thomas & Harden, 2008). In line with our aim of identifying and labeling specific time phases in the school reentry process, we followed three stages: (1) initial data familiarization, (2) identification of specific time phases and the related description within the examined review articles, and (3) final generation of a timeline with specific labels and consequent descriptions of the sub time phases. At each stage, two reviewers independently assessed the material, engaged in discussions, and reached a consensus.

The qualitative comments gathered during the Delphi Rounds 1, 2, and 3 were analyzed using qualitative content analysis, a systematic research method employed to examine textual data to identify patterns, themes, and meanings (Krippendorff, 2004). This method is commonly used in Delphi studies, and an inductive approach was applied (Belton et al., 2019). For each Delphi round, all the comments for each time phase were grouped, and a researcher conducted an initial read-through, making notes of keywords. Based on these keywords, a set of actions were identified. Each action would develop through the different time phases. At the intersection of each action with a specific time phase, respondents were invited to suggest one or more corresponding actions intended to facilitate the school reentry process. Similar or equivalent actions were grouped under a single statement. Another researcher with relevant expertise in the psychoeducation of children and young people with medical or mental health needs independently analyzed the data using the same procedure to assure confirmability. Where there was a discrepancy between the two researchers about a decision on how to classify a specific statement, the researchers discussed the matter to resolve the disagreement. If consensus was not achieved, a third author (SB) arbitrated.

### Participants

To recruit participants for the first round of the Delphi study, a list of 184 authors’ emails was generated from the authors’ lists of articles identified in the initial meta review (see Supplementary File 1). Invitations to register their interest in participating in the Delphi study were sent out, resulting in 42 experts responding positively. Subsequently, these experts were invited to participate in the first round, with 28 taking part and 23 completing the online questionnaire with valid data.

The second round was conducted in person at a pre-conference meeting of the 12^th^ HOPE Congress. Invitations were sent to 278 congress registrants, with 32 attending the Delphi meeting.

For the third round, invitations were extended to all participants from Rounds 1 and 2. Of these, 29 completed the round three questionnaire.

Overall, 56 participants from 18 different countries participated in at least one round of the Delphi study, with 24 experts taking part in two rounds (mostly Rounds 1 and 3, or 2 and 3) and two experts participating in all three rounds. Details for each round are provided in Table 1.

**Table 1:** Demographic details and professional experience of the participants in the three rounds of our Delphi study.


	ROUND 1	ROUND 2	ROUND 3

**Participants**	23	32	29

**Sex:**			

Female	78%	72%	76%

Male	22%	28%	24%

**Age:**			

Mean age (SD)	45 (14.80)	52 (7.98)	47.69 (14.15)

Age range	26–76	29–64	28–76

**Field Experience:**			

Physical Health Experts	16	17	13

Mental Health Experts	7	15	16

Mean Years of Experience (SD)	12.65 (9.56)	20.37 (11.65)	14.15 (11.29)

Range	1–40	5–39	1–40

Field of Work*:			

Education	11	25	14

Academia/Research	8	6	10

Health Care	4	1	4

Other	0	0	1

**Countries**	Australia Austria Canada Ireland Italy Namibia Slovenia Spain Switzerland United Kingdom USA	Australia Austria Belgium Finland Greece Hungary Italy Netherlands Romania Switzerland United Kingdom USA	Australia Canada Ethiopia Finland Greece Italy Namibia Netherlands Spain Switzerland USA


*Note*. *“Health Care” includes nurses, doctors, Child Life Specialists and other health staff. “Education” includes primary and secondary hospital and mainstream schoolteachers, principals, and education liaison. SD = Standard Deviation.

## Findings

### Round 0 – Rapid Meta Review

**Figure 2:**
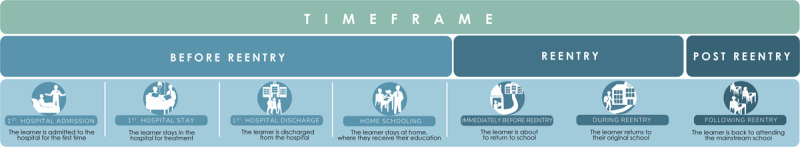
The three main timeframes and subsequent time phases of school reentry initially identified through the rapid literature review.

The initial review identified 15 articles addressing the school reentry process, including four focused on mental health and 11 on somatic illnesses (details provided in Supplementary File 1). Two of the present authors scrutinized these articles to identify key time steps relevant to a comprehensive school reentry process. This analysis, combined with the authors’ expertise in the field, led to the identification of three main timeframes (before-, at-, and post-reentry), which were further divided into seven specific time phases, as depicted in Figure 2. These findings served as the initial input for Delphi Round 1.

### Round 1 – Policy Delphi

The 23 experts involved in this round did not reach a satisfactory agreement (IRQ: 3.5; mean: 4.26; SD: 2.04) on whether the time-phase model accurately represents the key stages to be considered by professionals when facilitating the school reentry of learners with a medical or mental health condition after a prolonged school absence. However, one of the main reasons for this disagreement was that such initial time-phase models did not specify the actions to be undertaken, making their appropriateness difficult to judge. Gathering suggestions for such actions formed part of the next section of this Round 1 policy Delphi questionnaire. Therefore, this round resulted in 133 statements from physical health experts and 137 statements from mental health experts, covering all seven time phases identified in Round 0. The subsequent content analysis of these statements suggested an initial common set of 12 primary actions for the school reentry process: Welcoming, Consent, Communication, Support Networking and Empowering Connections at all Levels, Tailored Lesson Planning, Delivery and Reporting, Assessing, Monitoring, Supporting, Coordinator/Point Person, Multidisciplinary Care Team, Explaining the Disease and its Management in School, Developing Reentry Plans, Psychological Support, and Holding Meetings. Each action was enriched with descriptors and potential actions for each time phase. All these results are reported in Supplementary File 2.

This round led to the creation of a USRM, which included a final matrix of the 12 actions outlined above organized into seven time phases, and an additional section detailing the specific tools and activities. At this point, the USRM was identical for mental and physical health students and was used as the input for the Round 2 Delphi review session.

### Round 2 – Face-to-Face Expert Delphi Review

As anticipated in the methods section, the 32 experts who participated in the face-to-face Delphi review recommended the development of two distinct versions of the school reentry model: one tailored for learners with mental health needs and another for learners with physical health needs. The rationale for this recommendation was that although both models would share certain common elements, they must diverge in key areas. For example, the strategies for fostering connections with classmates and the division of time phases would differ between the two target populations.

A total of 52 comments were gathered from physical health experts: 12 addressed actions deemed inappropriate, 29 recommended additions to the model, and 11 offered practical activities and tools. Following analysis by the Delphi team, these comments led to 38 revisions and 14 rejections. Common reasons for rejections were that the suggestion was already present elsewhere in the model, the suggestion had been criticized by other groups with valid reasons, or that the comment did not contain any valid requests.

Similarly, mental health experts produced 135 comments: 31 concerned actions deemed inappropriate, 74 suggested additions to the model, and 30 contained suggestions for practical activities and tools. These results were analyzed by the Delphi team and led to 85 edits and 50 rejections, with reasons similar to those employed for the physical health model. Additionally, the “homebound education” time phase was removed from this model, and the “1^st^ hospital discharge” and “Immediately before reentry” phases were merged, because comments indicated that learners with mental health needs rarely have to stay at home after hospitalization.

These two school reentry models were termed the Physical Health – School Reentry Model (Physical H-SRM) and the Mental Health – School Reentry Model (Mental H-SRM). These models were then advanced to Delphi Round 3 for further refinement and to reach a consensus among the experts.

### Round 3 – Consensus Delphi

The third and final Delphi round took place online between January 25 and March 31, 2024, with the participation of 29 experts. This round utilized two separate questionnaires based on the two models generated by Round 2. Participants were instructed to respond to the questionnaire that best aligned with their expertise while maintaining an international perspective and avoiding a narrow focus on their specific context.

For the Physical H-SRM, the expert panel reached strong and consistent agreement on all the time phases and the model (see Table 2). There was moderate disagreement regarding the use of exclusively qualitative or quantitative measures to assess readiness for school reentry, with some experts believing that a combination of both approaches should be used. Finally, there was strong disagreement about the usefulness of applications and game solutions that present the disease as a battle to be fought (Table 2).

**Table 2:** Expert agreement (IRQ), mean Likert score (Mean), and standard deviation (SD) regarding; i) each action of the Physical HSRM, ii) the overall model (Overall), iii) the use of quantitative, qualitative, or a combination (Both) of approaches to assess school readiness, and iv) the use of applications and games that present the disease as a battle to be fought.


AGREEMENT FOR ACTIONS OF THE PHYSICAL H-SRM										

	1. WELCOMING	2. INFORMED CONSENT	3. EMPOWER COMMUNICATION AND CONNECTIONS	4. TAILORED LESSON PLANNING, DELIVERY, AND REPORTS	5. ASSESS, MONITOR, AND SUPPORT	6. COORDINATOR/POINT PERSON	7. MULTIDISCIPLINARY CARE TEAM	8. EXPLAIN THE ILLNESS AND ITS MANAGEMENT IN SCHOOL	9. PSYCHOLOGICAL SUPPORT	OVERALL

Mean	6.6	6.6	6.8	6.3	6.4	6.4	6.7	6.4	6.4	6.4

SD	0.5	0.6	0.6	1.3	1.1	0.6	0.5	0.8	0.8	0.9

IRQ	1	1	0	1	1	1	1	1	1	1

	PHYSICAL H-SRM SCHOOL READINESS ASSESSMENT METHODS AGREEMENT			AGREEMENT ON REPRESENT DISEASE AS A BATTLE						

	QUANTITATIVE	QUALITATIVE	BOTH							

Mean	5.6	6.1	6.5	3.9						

SD	1.2	1.1	0.8	2.1						

IRQ	2.3*	1.3	1	4*						


* Indicates lack of agreement.

This round also generated 57 comments related to actions 1–9, which are to be implemented by teachers and subsequently carried out with learners who have a physical illness. The analysis of these comments led to 33 rejections and 16 minor edits of the model. An additional eight comments were classified as remarks expressing praise or general agreement with the actions. Common reasons for rejection included the comment addressing actions already covered in the model (sometimes in different areas) or focusing on aspects related to specific roles or tasks that were deemed only relevant to the respondent’s context. Regarding the eight additional general comments on this model, five were coded as general praise and acknowledgment for the Delphi team. One comment outlined the need to perform a risk assessment for the young person to develop the model in a context-specific and flexible way and was included in the model; one comment related to the Delphi methodology, stating that the respondent would have preferred to evaluate every single item of each action of the model instead of all the items together grouped under the same action. One comment was used to expand upon an answer already given in a previous section of the questionnaire.

For the Mental H-SRM, the expert panel reached strong and consistent agreement on almost all the time phases, except for the contents of action 6 regarding “Coordinator/Point Person” for which agreement was only slightly above the threshold; however, the overall model received a strong consensus (see Table 3). Like the Physical H-SRM, the experts could not achieve a consensus regarding the usefulness of applications and game solutions that present the condition as a battle to be fought in the case of mental health issues.

**Table 3:** Experts’ agreement (IRQ), mean Likert score (Mean), and standard deviation (SD) regarding, i) each action of the Mental H-SRM, ii) the whole model (Overall), iii) the use of quantitative, qualitative, or a combination (Both) of school readiness assessment approaches, and iv) the use of images or software applications and games that present the condition as a battle to be fought.


AGREEMENT ON ACTIONS OF THE MENTAL H-SRM										

	1. WELCOMING	2. INFORMED CONSENT	3. EMPOWER COMMUNICATION AND CONNECTIONS	4. TAILORED LESSON PLANNING, DELIVERY, AND REPORTS	5. ASSESS, MONITOR, AND SUPPORT	6. COORDINATOR/POINT PERSON	7. MULTIDISCIPLINARY CARE TEAM	8. EXPLAIN THE ILLNESS AND ITS MANAGEMENT IN SCHOOL	9. PSYCHOLOGICAL SUPPORT	OVERALL

Mean	6.5	6.5	6.3	6.2	6.1	5.7	6.3	6.2	5.9	6.5

SD	0.5	0.7	0.9	1.1	1.1	1.7	0.8	1.1	1.3	0.5

IRQ	1	1	1	1	1	2	1	1	1	1

	MENTAL H-SRM SCHOOL READINESS ASSESSMENT METHODS AGREEMENT			AGREEMENT ON REPRESENT CONDITION AS A BATTLE						

	QUANTITATIVE	QUALITATIVE	BOTH							

Mean	6	6.4	6.7	3.5						

SD	1.2	0.5	0.5	2.1						

IRQ	1	1	1	3*						


* Indicates lack of agreement.

This round also generated 67 comments. Content analysis rejected 22 of these comments, and 39 resulted in minor modifications to the model. Additionally, six comments were categorized as expressions of praise or general agreement. The reasons for rejection were consistent with those applied to the physical illnesses model. Regarding the additional final comments for learners with mental health needs, three highlighted the necessity for the model to always be considered adaptable in terms of age-specificity and contextual relevance, stressing that not all interventions are automatically universally applicable or beneficial for all, and were included in the model. Two comments expressed general praise, with one also suggesting that the model could inform policy within health and education systems. One comment noted content already presented in one or more actions and time phases. Other sentences within these comments also indicated that the model should specify tasks rather than designate who should perform them and that the model was too complex.

Overall, the experts considered both models we proposed as representative of a valid school reentry process. Additionally, the experts recognized the benefits in both models of using a combination of qualitative and quantitative approaches (i.e., standardized and comparable) to assess school readiness. Finally, the use of apps and games that promote the idea of a “battle to be won or fought” polarized the experts, suggesting that such solutions are generally not well-received by professionals.

**Figure 3:**
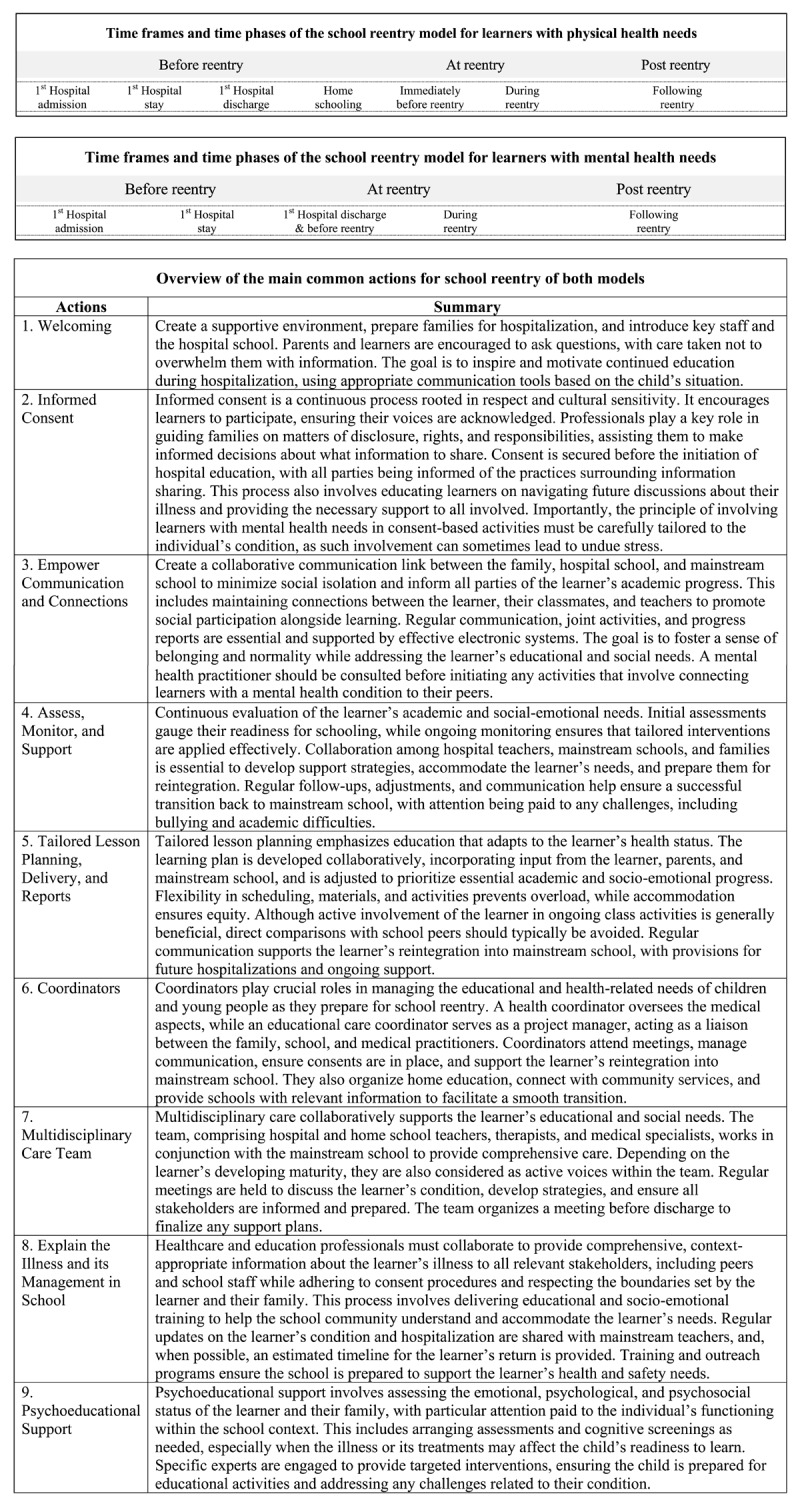
Overview of the time phases and actions in the final school reentry models for learners with physical or mental health needs. See Supplementary files for more details. Note: See Supplementary Files 3 and 4 for a high resolution and complete version of both reentry models.

After making minor edits, primarily involving the rearrangement of content within the actions and the substitution of more appropriate terms, the steering team finalized the two models of school reentry, as summarized in Figure 3 (for the complete models, see Supplementary Files 3 and 4).

The Physical H-SRM consists of the same seven phases proposed in the first round and includes nine actions. While the sequence of time phases is shown as consecutive, all actions listed under the same time phase should be considered contemporaneous (see Supplementary File 3). Moreover, to make the model more compact, actions that are cross-sectional and span more than one time phase are listed at the beginning of each action line. Due to the complexity within a set of different nested and mutually interacting systems (Plsek and Greenhalgh 2001) our model outlines functions and relationships rather than specific roles. Different actions deal with relationships at the learner’s and at the staff levels (i.e. Welcoming; Empower Communication and Connections; Coordinators); others deal with organization (Coordinators, Multidisciplinary Care Team), and some deal specifically with the education system (Tailored Lesson Planning, Delivery, and Reports, Assess, Monitor, and Support, Explain the Illness and its Management in School, Psychoeducational Support). Finally, one focuses on the learner’s and family’s rights to have their say in choosing when and how to share information (Informed Consent).

The structure of the Mental H-SRM is similar, with the same main actions. One of the main differences is that the Mental H-SRM model includes only five time phases (see Supplementary File 4). Specifically, home education has been removed as a specific phase since the experts’ comments indicated that this situation is rarely applicable to learners with mental health needs. Consequently, the 1^st^ Hospital Discharge and Immediately Before Reentry phases have been merged because they coincide.

Both models are preceded by three important recommendations, underscoring the need for contextualized, individualized, and collaborative approaches when implementing any specific action.

## Implications for Practice

The Mental and Physical H-SRM models are the products of an international study involving experts from diverse education and health fields across various countries. The models represent a reasonable synthesis of practices and suggestions found to be relevant by experts based on their experiences. Adaptations will be necessary when attempting to apply the proposed models to a local context. The models tend to propose actions and activities that can be broadly applicable but might clash with local cultural or organizational aspects and might require some contextual adaptations. Therefore, the local educational and healthcare teams must tailor the models’ recommendations to the specific cultures of the local school, hospital, associated families, learners, and the needs of other stakeholders. For similar reasons, practitioners and educators might find some of the terms used to be uncommon. The lack of a common terminology was one of the challenges faced by the Delphi team. Even experts from the same country sometimes used different words to express similar tasks or concepts. Additionally, the same terms are also evolving on a historical basis. The proposed models attempted a synthesis that can be used to enable or facilitate comparative analysis at a global level while maintaining the current terminology at a local level. The local professionals must understand the key concepts that a specific role or action entails and find the best way to translate those into local practices. For similar reasons, it is impossible to identify who should carry out a specific action. Education and healthcare systems vary depending on the country, the political model in government, and the international, national, and local socio-economic status. However, the key does not lie in delegating a specific task to a single role or function, rather, it lies in a multidisciplinary approach. For example, assessing school readiness (a crucial element of our model) should involve the medical team, which, depending on the mental or physical nature of the illness, should assess the possibility of the learner returning to school without posing any health risks to themselves or the community. At the same time, such a decision should involve the teachers, the hospital/home school, and the original school, who should assess the emotional and cognitive readiness of the learner to return, as well as the school’s and class’s readiness to welcome their schoolmate back. Finally, and most importantly, it should involve (and prepare) the learners and their families.

The two final models include numerous actions to be implemented across different time phases and considering them as a whole can be overwhelming. Like models from other fields (e.g., Federici, Scherer, & Borsci, 2014), it might be helpful for practitioners to view these not as prescriptive models but rather as ideal templates or frameworks. These can serve as reference points to guide action. The content of the models should be interpreted as a general overview of various areas to address, spanning the period from initial hospitalization to final school reentry. Not all schools and practitioners will be able to adopt all the recommendations simultaneously. Some suggestions may prove too expensive or impractical for certain individuals or contexts. However, given the richness of the models, it is likely that each school and healthcare team will find some actions that they are already implementing and others that they might initiate in the coming months. The recommendation is to start with these areas and engage in discussions within the school and multidisciplinary teams to determine which actions are most likely to yield the best cost-benefit in a specific context. For instance, social play or sport-based activities are increasingly being marginalized in schools and hospitals (Everett, Hubbuck, & Brown, 2023), yet they can often be reintroduced with little or no expense, involving volunteers, parents, or even peers (Dickey, Castle, & Pryor, 2016).

While the most apparent application of the models is to guide the various actions across different phases of a learner’s educational journey in a hospital school, ensuring the necessary adaptations, they can also be applied at multiple levels.

Specific actions or time phases could easily generate content for interactive workshops or training activities to be carried out with practitioners and school personnel. For example, doctors, nurses, teachers, volunteers, and any other health care practitioners could discuss their own welcoming practices for newly hospitalized children and young people and compare them with the actions and suggested activities from the Mental and Physical H-SRMs.

The models could potentially serve as a foundational document to inform policy within health and education systems. This is particularly crucial in the context of hospital schools, where policies must address the complexity and diverse areas outlined in the model. Effective policy-making should provide foresight in planning clear roles and functions, and ensure the provision of adequate resources, including appropriate working hours, task allocations, and specialized training that goes beyond the typical requirements for ordinary teachers. It should also require medical and educational coordinators to oversee the provision of adequate education throughout the different time phases and organize the school reentry process. Finally, a good policy should provide for as well as sustain ongoing teamwork and supervision.

Moreover, these models could offer valuable insights for those designing teacher training curricula, particularly for hospital educators. By highlighting critical areas of expertise, the models can guide the development of training programs that equip hospital teachers with the skills and knowledge necessary to meet the unique challenges of educating students in medical settings. This specialized training would enhance the quality of the education provided in hospital schools and ensure that teachers are well-prepared to support the complex needs of their students.

Even though Delphi is a valid and widely used technique in research on healthcare and related services, this method has some limitations (Keeney et al., 2011). The iterative and sequential nature of a Delphi study can be lengthy; our three rounds spanned over 16 months. While this approach allowed us to reach participants from several different countries and organize a face-to-face meeting, the workload may have been overwhelming for some, causing fatigue. This could have led to incomplete or rushed responses from some participants and may also explain the dropouts.

In the spirit of the Delphi method, it was considered valuable to gain insights from various countries to ensure a broad perspective on school reentry. However, the final models aim to summarize and standardize, rather than emphasize the diversity and richness of different approaches. Consequently, the final models might not be suitable in certain contexts or for medical reasons in specific situations. Typical examples are activities aimed at improving connectedness between the learner and the school. While the literature highlights the many beneficial effects of such interventions (Tomberli & Ciucci, 2021), there may be times in the therapeutic journey when this could be deemed inappropriate by the treatment team. For instance, in certain mental health cases, such as Social Anxiety Disorder, Oppositional Defiant Disorder, or Agoraphobia, the prospect of facing a class, even remotely, can exacerbate feelings of anxiety, aggression, or social withdrawal. In such situations, while reconnecting with school remains a critical milestone for developmental progress and recovery, the timing and modality of this reintegration may need to be adjusted according to specific medical recommendations. Additionally, it is essential to appropriately prepare the learner’s class of belonging to ensure a supportive environment.

A key limitation of this Delphi study is the absence of input from parents and learners, despite the recognition that they are the true experts of their own experiences. Various models emphasize the importance of including these voices throughout the research process (Keeney et al., 2011). However, involving learners with medical or mental health conditions and their families in a Delphi study presents significant challenges. These include concerns about privacy, the instability associated with ongoing medical conditions, and the extended duration of the study, which spanned over two years. While these reasons made it impractical to involve learners and their families in this study, such involvement is crucial, frequently emphasized in various actions in our models, and should be pursued at the local level. Local involvement can be facilitated through methods such as focus groups, group work, reflective practice groups, student councils, and engagement with local parent associations, all of which can ensure that these essential voices are heard and considered (Mockler & Groundwater-Smith, 2014).

## Conclusion

The findings from this Delphi study provide a comprehensive framework for supporting the school reentry of learners with medical or mental health conditions. The development of the Physical H-SRM and Mental H-SRM models offers structured guidance for educators, healthcare providers, and families, emphasizing the importance of a multidisciplinary and context-sensitive approach. The study highlights the critical role of tailored interventions across distinct time phases, from hospitalization to full reintegration into the school environment. While the models are robust and reflect international expert consensus, their successful application will always require careful adaptation to local contexts and cultures. This research highlights the complexity of school reentry, advocating for collaborative strategies that consider the unique needs of each learner and their environment.

For children and young people with medical or mental health conditions, school reentry presents a journey filled with challenges, opportunities for personal growth, and the development of relationships across various contexts. By viewing the entire process through a systemic lens, we believe that our models can play crucial roles in fostering resilience and promoting active involvement from all stakeholders throughout each phase of the journey, thereby supporting the well-being and continued progress of everyone involved.

## Additional Files

The additional files for this article can be found as follows:

10.5334/cie.159.s1 Supplementary File 1. Summary of the results of the Rapid Meta Review.

10.5334/cie.159.s2 Supplementary File 2. Unified School Reentry Model (USRM) developed from Delphi Round 1 and distributed to all participants in preparation for Round 2.

10.5334/cie.159.s3 Supplementary File 3. Poster with the Physical H-SRM.

10.5334/cie.159.s4 Supplementary File 4. Poster with the Mental H-SRM.
