# Supplementary material for: Pathways to School Reentry for Children and Young People with a Medical or Mental Health Condition: An International Delphi Study
Source: Contin Educ. 2025 Mar 5;6(1):38–57. doi: 10.5334/cie.159 (PMC11887473; doi:10.5334/cie.159)
Supplement: Supplementary File 1. — Summary of the results of the Rapid Meta Review. [file cie-6-1-159-s1.pdf]

Pathways to school reentry for children and young people with a medical or mental health condition: an international Delphi study

Supplementary Material 1

Capurso, Moracci, Borsci

Rapid Meta-Review of Literature on School Reentry for Children and Young People with Mental Health Needs

Autor, Year	Title	Main objectives	Nr. of examined Articles	Main findings
Cooper, Evans, & Pybis, 2016	Interagency collaboration in children and young people's mental health: A systematic review of outcomes, facilitating factors, and inhibiting factors.	To evaluate the outcomes of interagency collaboration within child and youth mental health services, while also identifying the factors that may either support or hinder this collaboration.	33	The factors most frequently identified as promoting interagency collaboration included robust communication between agencies, joint training programs, a clear mutual understanding, reciprocal respect, support from senior management, established collaboration protocols, and the appointment of a dedicated liaison.<br>Conversely, the most commonly cited obstacles to interagency collaboration were limited resources, poor communication between agencies, lack of mutual respect, differing viewpoints, insufficient understanding across agencies, and concerns about confidentiality.
Midura, Fodstad, White, Turner, & Menner, 2023	Supportive Transition Planning for Adolescents Transitioning From Psychiatric Hospitalization to School: A Systematic Literature Review and Framework of Practices	To collect and synthetize evidence from the literature in the areas of barriers, challenges, and significance of the need for a formal transition planning framework in order to provide an accessible real-word framework and template for practitioner use.	24	Four major key factors emerged as critical for developing a transition planning framework from acute psychiatric hospitals to school-based settings: (a) Stakeholder Voice (including the perspectives of the Student, Caregiver, Hospital/Treatment Team, and School Team); (b) Establishing a Point Person for Transition (either from the Medical or School side); (c) Recommendations/Accommodations (encompassing both formal and informal supports); and (d) Conducting a Transition Meeting.
Tougas, Rassy, Frenette-Bergeron, & Marcil, 2019	"Lost in transition": A systematic mixed studies review of problems and needs associated with school reintegration after psychiatric hospitalization	To identify, through a bioecological perspective, the problems and needs of the different actors concerned by the issue of school reintegration after psychiatric hospitalization.	14	At a conceptual level, the results affirm the importance of understanding school reintegration as an ecological transition process that begins on the first day of hospitalization and continues after the student returns to school.<br>In terms of individual problems and needs, the results indicate a high degree of complexity. When managing complex systems such as families, schools, and healthcare, an individualized intervention approach combined with a comprehensive analysis of specific environmental situations appears to be more appropriate. While the results highlight problems and needs unique to each environmental situation

				and configuration (microsystems and mesosystems), they also underscore overarching themes such as communication, collaboration, and coordination.
Tougas, Houle, Leduc, Frenette-Bergeron, & Marcil, 2023	Framework for Successful School Reintegration after Psychiatric Hospitalization: A Systematic Synthesis of Expert Recommendations.	To identify and synthetize expert recommendations from best available clinical and scientific literature for successful school reintegration of students after psychiatric hospitalization and to define essential guidelines for practice.	53	A nine-step framework translating expert recommendations was established:<br>1 - Address school reintegration as soon as possible: Emphasizes the importance of initiating the school reintegration process early by fostering collaboration and information sharing among all stakeholders involved in the hospitalized student's life.<br>2 - Evaluate needs during hospitalization: Involves the continuous assessment of the student's needs concerning school reintegration to develop a shared understanding of both the current and desired situations during their psychiatric hospitalization.<br>3 - Offer academic and psychosocial support during hospitalization: Occurring concurrently with Step 2, this step recommends implementing concrete actions to support the student's academic progress and psychosocial interventions to assist the student and their parents in preparing for school reintegration.<br>4 - Ensure leadership in the coordination of a school reintegration plan: Involves designating a liaison to act as a coordinator responsible for developing and overseeing the school reintegration plan and its follow-ups.<br>5 - Evaluate needs in anticipation of the student's return to school: Focuses on assessing the overall functioning and needs of the student, their parents, and school stakeholders in preparation for the student's return.<br>6 - Develop a school reintegration plan: Involves creating a school reintegration plan that includes recommendations for optimally supporting the student's transition back to school.<br>7 - Intervene alongside the social environment: Entails implementing interventions before discharge to ensure the school environment is prepared to accommodate the returning student.<br>8 - Intervene alongside the student and their family: Involves providing the necessary support to the student during their return to school, agreeing on a safety net, and ensuring the successful implementation of the reintegration plan.<br>9 - Complement the reintegration plan: Involves informing the follow-up partners about the reintegration plan and mobilizing them as necessary for its implementation and revision.

This document contains supplementary material for the above-mentioned article, as provided by the authors.
The original article can be downloaded from https://doi.org/10.5334/cie.159

Rapid Meta-Review of Literature on School Reentry for Children and Young People with Physical Health Needs

Autor, Year	Title	Main objectives	Nr. of examined Articles	Main findings
Barnett, Tollit, Ratnapalan, Sawyer, & Kelaher, 2023	Education support services for improving school engagement and academic performance of children and adolescents with a chronic health condition	To perform a Cochrane Systematic Review To describe the nature of educational support interventions for children and adolescents with a chronic health condition, and to examine the effectiveness of these interventions on school engagement and academic achievement.	4	Many of the reviewed studies revealed significant limitations or were plagued by methodological flaws. Specifically, numerous studies failed to report trial results, thus not meeting the inclusion criteria for this Cochrane Systematic Review, which required studies to be controlled trials or interrupted time series studies. As a result, a meta-analysis was not feasible due to the insufficient number of studies per outcome, which would have impeded the accurate estimation of between-study variability necessary for a random effects meta-analysis.<br>There was some evidence suggesting that educational support improved school engagement, with three out of four studies favoring the intervention. However, the overall certainty of this evidence was judged to be very low. Regarding academic achievement, three of the four studies measured this outcome, but only two provided effect estimates, yielding contradictory results.<br>A single study examined the impact of educational support on the transition back to school, finding a positive effect in favor of the intervention. However, this study had a small sample size (n = 30), and the confidence interval suggested the possibility of a very small or negligible effect.<br>Two of the four studies assessed self-esteem, with both reporting positive effects from educational support interventions. This was the only outcome for which the overall certainty of evidence was judged to be low rather than very low, suggesting some evidence that educational support may slightly improve mental health as measured by self-esteem.<br>No studies reported measures of quality of life or adverse outcomes.<br>The authors concluded that the quality of research on the effectiveness of educational support interventions for children and adolescents with chronic health conditions remains in its infancy. At best, there is uncertainty regarding whether these interventions improve academic achievement or school engagement. Similarly, the evidence is uncertain regarding their impact on transitions back to school or school re-entry. However, there is some evidence that educational support may slightly improve self-esteem.
Canter & Roberts, 2012	A systematic and quantitative review of interventions to facilitate school reentry for children with chronic health conditions.	To conduct a systematic and quantitative review of research on the effects of school reentry interventions for children with chronic health conditions.	12	The results support the effectiveness of school reentry programs in increasing specific knowledge and fostering positive attitudinal changes in schools. Overall, the impact on knowledge was more substantial compared to the effect on positive attitudinal change. Moreover, interventions aimed at teachers produced larger effects in both knowledge acquisition and positive attitudinal change compared to interventions targeting healthy peers.
Castro Ibáñez, 2023	The education of children in hospital schools. a literature review.	To find evidence in the literature to describe and characterize the pedagogical practices developed in the	32	There is no established model of pedagogical practices specifically designed for students educated in hospitals; rather, there are only suggestions for their implementation.<br>Three types of Regulatory Framework for pedagogical practices are reported. The first involves the legal recognition of a school or classroom located within the hospital. The second pertains to practices that occur

This document contains supplementary material for the above-mentioned article, as provided by the authors.

The original article can be downloaded from https://doi.org/10.5334/cie.159

		educational context of the hospital in terms of their regulation, format, duration, content, and associated benefits.		outside formal recognition or regulation. The third involves practices developed under regulations that emerge from research projects or educational interventions.<br>Hospital Teachers: Pedagogical practices are not always conducted by education professionals. However, professional training alone is insufficient; teachers must also possess a range of skills related to emotional and social intelligence.<br>Student-Patients: Educational care is provided at various stages, such as pre-school, elementary school, or high school, with each program adapted to the student's learning process.<br>Two types of classrooms are reported. The first is a traditional classroom, where lessons are held in a room designated specifically for this purpose within the hospital premises. The second type is the hospital bedroom, where classes are conducted directly at the students' bedsides.<br>Duration: The time allocated for pedagogical activities ranges from half an hour to four hours per day per student.<br>Teaching in hospital settings, whether in multigrade classrooms or directly at the bedside, consistently adheres to the principle of flexibility and adaptation to the individual learning needs of each child or young person.<br>Three types of benefits, or "positive effects," of educational care for children and adolescents in hospital settings were identified: benefits for the medical situation, benefits for the educational situation, and additional benefits.
Helms et al., 2016	Facilitation of school re-entry and peer acceptance of children with cancer: A review and meta-analysis of intervention studies.	The aims of this study were to: (1) review and analyse the existing literature on school re-entry interventions for children with cancer; and (2) discuss the importance of peer involvement in the treatment.	6	The meta-analysis results support the efficacy of school re-entry programs in enhancing academic achievement and reducing levels of depression in children with cancer. However, no significant effects were observed in terms of reducing behavioral problems or improving social competences.<br>In reviewing peer-support programs, it was found that these programs significantly increased classmates' knowledge about cancer. This increase in knowledge was associated with reduced fear and a more positive attitude toward the child with cancer. Additionally, the studies reported a greater willingness among classmates to interact with the child with cancer and a decrease in their personal concerns.
Lindsay et al., 2015	A systematic review of hospital-to-school reintegration interventions for children and youth with acquired brain injury.	To perform a literature review on interventions that aimed to improve hospital-to-school reintegration for children and youth with acquired brain injury (ABI).	17	The findings indicate that school reintegration interventions for youth with moderate or severe acquired brain injury (ABI) have the potential to enhance knowledge of ABI, cognitive functioning, behavior, problem-solving skills, social skills, and coping abilities. However, there is minimal emphasis, if any, on educating teachers or peers.<br>The results also suggest that effective hospital-to-school reintegration interventions may consist of several different components. Common elements of successful interventions include one-on-one sessions led by a trained clinician or educator, homework activities, and parental involvement. Additionally, other multimedia methods and materials—such as videos, art, games, and role-playing—may be valuable strategies to support youth in transitioning back to school. Younger children may benefit from storytelling or puppet use, while older youth might find peer mentor support particularly advantageous.
Lum et al., 2017	Understanding the school experiences of children and adolescents with	(1) Examine the school experiences and outcomes of children and adolescents with chronic illness,	18	Consistent evidence was found linking chronic illness with lower attendance rates. The findings related to academic, interpersonal, and behavioral domains were mixed. However, investigations into students' engagement and reintegration experiences revealed several challenges amidst generally positive experiences. Poorer school experiences and outcomes were consistently associated with greater disease

This document contains supplementary material for the above-mentioned article, as provided by the authors.
The original article can be downloaded from https://doi.org/10.5334/cie.159

	serious chronic illness: A systematic meta-review.	emphasizing findings from high-quality reviews, (2) Examine the association between illness-related medical, school, psychosocial and sociodemographic factors and school experiences and outcomes, (3) Provide recommendations for educators, health professionals and researchers.		severity, stronger treatment side effects, and lower socioeconomic status (SES). Effective models of school support were repeatedly linked to school success among students with chronic illness.<br>The results underscore that school support is strongly associated with better school experiences and outcomes for students with chronic illness, including the use of homebound education, school reintegration programs, and individualized education plans.<br>In terms of practical recommendations, this meta-review emphasized the wide range of challenges associated with chronic illness in the school environment, highlighting the need for school support that addresses academic, social, emotional, and physical needs. A coordinated and collaborative approach involving educators, healthcare professionals, psychologists, and the student and their family is essential to meet this diverse set of needs.
Pan et al., 2018	School reintegration of pediatric burn survivors: An integrative literature review.	The objective of this study was to identify, summarize, and integrate current knowledge on school reintegration for pediatric burn survivors.	13	The results indicate that school reintegration following a burn event is a process that begins early after hospital admission and is customized to the individual child and their specific circumstances. Three key participants were identified in the school reintegration process: the child, the parents, and the teacher.<br>The analyzed studies did not show a significant effect on the child's overall adjustment; however, they did suggest that children who participated in the program experienced a faster return to school.<br>Three phases were identified, each with distinct needs that guide the type of support required. In the first phase, the preparation phase, which begins during hospitalization, the focus is on informing the teacher and classmates. During this phase, the child and parents may need to address the physical and emotional consequences of the burn. Parents play a crucial role in maintaining contact between the child and the school, given their intimate knowledge of their child's needs and school background.<br>The second phase, the return to school, involves the actual school visit, which is considered a vital component of the program. During this visit, teachers and classmates are educated on a wide range of topics related to living with burns, including physical and psychological aspects, as well as issues specific to burn injuries.<br>The third phase, after the child has returned to school, encompasses the ongoing school reintegration period. During this phase, children may struggle with the physical aftermath of burns, such as scars and pressure garments, and may face unpleasant remarks about their altered appearance. This particular need appears to be inadequately addressed in current school reintegration programs.
Prevatt, Heffer, & Lowe, 2000	A review of school reintegration programs for children with cancer.	To review the literature on school reintegration programs for children with cancer identifying best practices.	14	School Personnel Workshops appear to increase educators' knowledge of both the medical and psychosocial aspects of childhood cancer, as well as enhance their comfort level when working with an ill child. These programs are typically brief, lasting a maximum of 8 hours, and can accommodate a large number of participants at once. However, a notable drawback is the lack of empirical evidence indicating that the benefits gained by educators are effectively transferred to the ill child.<br>Peer programs are cost-effective, reaching a large number of participants in a short period. Positive outcomes include increased knowledge of the medical and psychosocial aspects of cancer and greater interest in interacting with ill peers. However, evidence regarding the long-term effectiveness of these programs and the relationship between increased knowledge and peer acceptance is inconsistent.

This document contains supplementary material for the above-mentioned article, as provided by the authors.

The original article can be downloaded from https://doi.org/10.5334/cie.159

				Comprehensive Programs lack a solid theoretical foundation and a clear model for program implementation.
Tomberli & Ciucci, 2021	Sense of School Belonging and Paediatric Illness: A Scoping Review	To explore the literature on the relationship between pupils with medical condition and their classmates, particularly, their sense of school belonging (SoSB)	10	The findings indicate that a Sense of School Belonging (SoSB) is a fundamental psychological need for pupils with chronic health issues, and that technology can be an effective means of connecting these children with their class.<br>The recommendations suggest that both hospital and regular schools should actively create opportunities to connect pupils with chronic health issues to their classes, addressing both academic continuity and relational aspects (including formal and informal contacts). Interventions aimed at promoting SoSB should consider the following: (a) the availability of suitable technologies within the regular school to facilitate connections for the child with chronic health issues; (b) the user-friendliness of these technologies to ensure they are practical for school use; (c) the recognition that technology alone is insufficient; and (d) the pupils' perspectives on being connected to their class through appropriate technologies.
Vanclooster, Benoot, Bilsen, Peremans, & Jansen, 2018	Stakeholders' perspectives on communication and collaboration following school reintegration of a seriously ill child: A literature review.	To study the perspective of parents, school personnel and healthcare providers on communication and collaboration following the child's return to school, to gain insight into their experiences, and to develop recommendations for each stakeholder.	22	The primary topics of interaction among parents, school personnel, and healthcare providers are knowledge, education, and support related to the child's condition. However, their experiences indicate variable and often inadequate practices in communication and collaboration. They also report having complementary needs and expectations regarding effective collaboration, with all parties recognizing the value of a school liaison as a critical link between the family, education, and healthcare.<br>The results highlight a well-documented lack of knowledge within schools and a notable absence of initiatives to provide necessary information and training to school personnel. Furthermore, many peers at school are insufficiently informed about the child's condition, which can lead to interpersonal difficulties following the child's return to school.<br>The findings point to procedural issues that arise during the school reintegration process, particularly concerning the interaction between stakeholders. Communication involves not only direct personal contact but is also shaped by the broader context in which these individuals operate.<br>This review suggests that healthcare providers can play a constructive role in the school reintegration process. Beyond offering direct services to the child, health professionals are essential for providing accurate health-related information to parents and schools, as well as offering training or support opportunities when needed.
Wikel & Markelz, 2023	Chronic Health Conditions, School Attendance, and Socioeconomic Factors: A Literature Review,	To study the extent to which the field has examined chronic illness, chronic absenteeism, and poverty.	14	Students with chronic health conditions experience higher rates of school absences compared to their peers. The results also reveal that socioeconomic status (SES), when combined with chronic health conditions, significantly impacts school outcomes.<br>The chronic health conditions examined in the review included asthma, type 1 diabetes, kidney disorders, transplants, cancer, epilepsy, sickle cell anemia, pulmonary disorders, cardiac conditions, and others. Among these, asthma was identified as the most frequently studied chronic illness in relation to socioeconomic factors and school attendance.<br>The review suggests that parental education level and parental involvement in their children's education may play a more crucial role in cognitive development than economic factors alone.

Note: The bibliography can be found in the main article's reference section.

This document contains supplementary material for the above-mentioned article, as provided by the authors.

The original article can be downloaded from https://doi.org/10.5334/cie.159
