# Supplementary material for: Pathways to School Reentry for Children and Young People with a Medical or Mental Health Condition: An International Delphi Study
Source: Contin Educ. 2025 Mar 5;6(1):38–57. doi: 10.5334/cie.159 (PMC11887473; doi:10.5334/cie.159)
Supplement: Supplementary File 2. — Unified School Reentry Model (USRM) developed from Delphi Round 1 and distributed to all participants in preparation for Round 2. [file cie-6-1-159-s2.pdf]

Pathways to school reentry for children and young people with a medical or mental health condition: an international Delphi study

Supplementary Material 2

Capurso, Moracci, Borsci

Rapid Meta-Review of Literature on School Reentry for Children and Young People with Mental Health Needs

Part of the international Delphi study on *factors that facilitate school reentry of children and young people with a medical or mental health condition after prolonged school absence* run by the university of Perugia and the university of Twente

Dear Participant at the Consensus Conference on the school reentry of children and young people with a medical or mental health condition after prolonged school absence,

First of all, thank you for joining us and helping with this project! This conference is part of a wider Delphi study aimed at developing guidelines or a good school reentry of young students with a medical or a mental health condition.

This Unified School Reentry Model reports on the results of our previous round of the Delphi study. It shows the different phases that were identified in connection with a school reentry process and the related actions or recommendations.

During this meeting, you will be asked to comment on the content of this model. Do not worry, you will not have to work on the whole document! You will be organised in a small group and only get a few 'actions' to work on. However, **it is important to have an overview of all the actions** in order to avoid working on things that are already part of a different time-phase of the process.

The consensus conference will be held at Palazzo delle Stelline, Corso Magenta 61, Milan, on 9 May 2023, at 15.00 – 17.00 in the "Toscanini" room.

We look forward to doing this piece of work together!

Michele Capurso, Valentina Moracci, University of Perugia
Simone Borsci, University of Twente

	Before reentry				Reentry		Post reentry	Tools and activities
Phases / Actions	1st hospital Admission	1st hospital stay	1st hospital discharge	Homebound Education	Immediately prior to reentry	During reentry	Following reentry	
**Welcoming**	Offer an initial welcoming to facilitate a flexible, friendly, and supportive environment.<br>During the welcoming the school is presented to the parents and they are encouraged and motivated towards continuing their child education.<br>Department staff is presented to the patient and the carers and the department itself is explained to them.	Present the school to the pupil and parents as they are encouraged and motivated towards continuing their child education.	Facilitate a flexible, friendly and supportive environment.					Ward map.<br>"who is who" billboard with staff pictures and roles,<br>Creative identity card for the presentation of the pupil….<br>… *To be continued at the consensus conference*
**Consent**	Obtain consent in order to share relevant information in a respectful way.<br>Obtain consent from parents/carers and any other relevant stakeholders.<br>Consent for underage student can be obtained in verbal form.<br>The pupils with chronic illness should be involved in the whole process to express their point of view and have their voice heard, in a manner consistent with their evolving capabilities.	Have parent/guardian sign consent form to contact school/key stakeholders outside of the hospital system.			Talk to the pupil about how to describe their illness/condition to peers or how to redirect conversation if they don't want to talk about it.<br>The pupil with chronic illness should be involved in the whole process to express his point of view.	Listen to the student's voice.<br>The pupil with chronic illness should be involved in the whole process to express his point of view.		*To be done at the consensus conference*
**Communication**	Create a communication link between family-hospital-school in order to create a shared work plan and to minimize social isolation and school difficulties.<br>The hospital school should assess school needs and current functioning in order to develop a continuous education plan.	Collect academic data from the mainstream school and share to create activities that are in synergy between the different istitutions.<br>The hospital school can liaise with the mainstream school to advocate and support the student in staying engaged in the social and	The support and agreement with the pupil's mainstream school continues.<br>Give all the relevant information collected to the home teacher in order to become part	During home stay the support and agreement with the pupil's main school continues.<br>Maintain the link between family-hospital-school in order to minimize	Give relevant information about health conditions of the pupil and the precaution needed from the classmates.<br>Create a link between family-hospital-school in order to create a shared work plan and to minimize social isolation and school difficulties.	The hospital teachers should be available to the mainstream teachers to stay in touch and answer to their needs of understanding the pupil behaviour.<br>Strong peer groups should be created that are respectful of	Maintain link between family-hospital-school to nourish the shared work plan and to minimize social isolation and school difficulties.	*To be done at the consensus conference*

This document contains supplementary material for the above-mentioned article, as provided by the authors.

The original article can be downloaded from https://doi.org/10.5334/cie.159

Phases / Actions	1st hospital Admission	1st hospital stay	1st hospital discharge	Homebound Education	Immediately prior to reentry	During reentry	Following reentry	
	Collect academic data from the mainstream school and share to create activities that are in synergy between the different istitutions.	emotional aspect of development as well as educational.<br><br>Hospital teacher should act as a bridge between those involved to maintain constant and beneficial communication.<br><br>Favour a sense of school belonging between hospitalized children, classmates and teachers.<br><br>Create ongoing communication with the mainstream school, keeping them informed of the pupil's academic progress.	of the educational team.<br><br>Favour a sense of belonging between hospitalized children, classmates and teachers.<br><br>Maintain contact with the hospital school to nourish the teacher-pupil relationship.	social isolation and schooling difficulties.<br><br>The homebound teacher should communicate regularly with the mainstream teachers in order to make the teachings as relevant as possible to the educational needs.<br><br>Maintain contact with the hospital school to nourish the teacher-pupil relationship.<br><br>The hospital teacher should be available to be contacted to assist with homebound education.	Share academic and diagnostic information.<br><br>Hospital teacher communicates with the mainstream school regarding the student upcoming discharge from the hospital and return to mainstream school.	diversity, thus preventing or hindering bullying due to illness.<br><br>Maintain contact with the school in the hospital to nourish pupil-hospital teacher relationship.<br><br>Keep the communication between family, hospital and school regular.<br><br>Share academic and diagnostic information.		
**Support Networking and empower connections at all levels**	Create connections and empower sense of belonging.<br><br>Maintain the connection between the child and the mainstream school.<br><br>Favour a sense of belonging between hospitalized children, classmates and teachers.<br><br>Identify classmate designed to visit the hospitalized pupil.	Maintain connections and empower sense of belonging.<br><br>Encourage the school/classroom to communicate regularly with the diseased pupil.<br><br>Mainstream teacher should send updates about what is going on at school.	Maintain connections and empower sense of belonging.<br><br>Identify one or more classmates to visit the diseased pupil to update on the mainstream school work progress.<br><br>The support and agreement with the pupil's mainstream school continues.	Build strong peer groups that are respectful of diversity in order to prevent or hinder bullying due to illness.<br><br>Favour a sense of belonging between diseased pupil, classmates and teachers.	Perform a peer presentation when needed and if appropriate to pupil.	Promote social participation and continuous contact of the pupil with friends and peers.<br><br>Create a sense of normality in the life of the student to make it more similar to other students.	Promote social participation and continuous contact of the pupil with friends and peers.<br><br>Be aware that there might be bullying of the pupil because of the hillness.	Calls, video calls and any relevant technology that can be used to maintain contact.<br><br>*… To be continued at the consensus conference*
**Tailored lesson planning, delivery and report**	Once the voice of parents, students and medical staff have been heard (recommendations, questions, concerns), child priorities and motivations should be explored.	Collect baseline information from the student, the parents as well as the school regarding both concerns & strengths about school.<br><br>Once the school work plan is redacted the student gets lessons according to his condition (at the	The hospital teacher produces a report that states what has been done and where the pupil should get support once back home.	An hospital school report should be given to the teacher for home tuition of the pupil.<br><br>The mainstream school has to	An academic check should be performed to determine if the student is academically comparable to their peers.<br><br>Transfer ownership of needs and recommendations to school and local provider transition team.	Create a plan with school and teachers for future hospitalizations.<br><br>Promote individualized attention and education in order to guarantee equity.	The mainstream school should support the pupil if found behind on the academic program.<br><br>Promote individualized attention and education	*To be done at the consensus conference*

This document contains supplementary material for the above-mentioned article, as provided by the authors.

The original article can be downloaded from https://doi.org/10.5334/cie.159

Phases / Actions	1st hospital Admission	1st hospital stay	1st hospital discharge	Homebound Education	Immediately prior to reentry	During reentry	Following reentry	
	Hospital teachers and mainstream teachers cooperate in creating a plan for school work that is based on the information collected.<br><br>The plan should include only the workload deemed to be priority in the student's academic or social-emotional progress and will also account for possible future hospitalizations.	bedside for example) and following the mainstream school curriculum.<br><br>The plan should include only the workload deemed to be priority in the student's academic or socio emotional progress.<br>Alternative teaching/learning scenarios should also be explored encouraged and implemented with open and flexible formulas.	Give the report to the mainstream school and to the home liaison officer so that the home work can be informed.<br><br>The hospital school teachers should identify learning material in conjunction with mainstream school teachers and provide the student with school supplies, activities and encourage them to do schoolwork at home.<br><br>Home tuition can be organized by the mainstream school and carried out by different figures (eg. Home liaison officer)	organize home tuition for the pupil after receiving indication from the hospital school.<br><br>Determine if the pupil is academically comparable to peers.<br><br>If the academic level of the pupil is inferior to that of the peers plan actions to help reach the requested grade level.<br><br>Help family identify specialists for their needs related to issues/difficulties during initiation of home-based education.<br><br>Tackle an amended amount of schooling each day.<br><br>The parents should keep the pupil busy at home with the activities provided by the hospital or mainstream school.<br><br>The student can have school work during virtual lessons with mainstream teachers.	The pupil needs to be supported offering shorter days or revised timetable when needed.<br><br>Discuss parents rights and school responsibilities in serving students with special need/health conditions.<br><br>The head mainstream teacher should offer a place for discussion where to express feelings, worries, doubts.	Educational professionals should be aware and flexible towards what is happening and the needed adjustments.	in order to guarantee equity.<br><br>Promote independence and growth for the child as appropriate.<br><br>Teachers should set up flexible learning materials.	
**Assess, monitor and support**	Perform an assessment of the pupil's academic needs, strengths and challenges.	Once the stressors and risk factors are identified, the medical team and/or the hospital teacher will accompany the students/carers in identifying strategies and	Ensure homebound supports are adeguate.		Assess patient's and family's preferences/desires for school re-entry support.	Put the accommodations/modifications into place.<br><br>Monitor re-entry progress.	Provide a constant monitoring of the pupil's attendance trends and any problems that may have arisen at school.	*To be done at the consensus conference*

This document contains supplementary material for the above-mentioned article, as provided by the authors.

The original article can be downloaded from https://doi.org/10.5334/cie.159

Phases / Actions	1st hospital Admission	1st hospital stay	1st hospital discharge	Homebound Education	Immediately prior to reentry	During reentry	Following reentry	
	Perform an assessment of the pupil's/family's risk factors with validated instruments such as the Quality of Life Measure.	resources to overcome them and targeted supports are identified.<br><br>The accommodations/modifications are then put into place.<br><br>Support mainstream school for the eventual organization of home lessons.<br><br>Provide support to the student's family/parents/siblings as needed.	The school principal should assess the extent to which the school actors are ready to support the pupil's re-entry.<br><br>The medical team should assess the readiness of the child and family to be reintegrated into the mainstream school environment.<br><br>Parents start the formal process of adaptation of schooling of a student with a long term illness.		Identify the accommodations the pupil will need.<br><br>Hospital school teachers should talk to doctors and social workers to establish if the pupil is ready to return to school before they recommend it.	Have frequent check-ins with pupil, family and teachers on the reintegration process<br><br>Check if the interventions and supports put in place are working and if they are still desired.<br><br>Ask pupil if the needed accommodations are being applied and if they are satisfied with them.<br><br>If the pupil is having academic challenges refer for an assessment from a pedriatic neuropsychologist<br><br>Have a planned follow up on the transition plan with student and mainstream school staff.<br><br>Monitor plan/progress as needed and make due changes.<br><br>Teachers should provide the pupil with the needed support.<br><br>The clinical psychologist follows the student's re-entry process and cooperates with school and parents.	Regularly assess re-entry experience with both the pupil and the family.<br><br>After an assessment of the re-entry experience adjust support to family and child in real time.<br><br>Identify the practices that were successful in handling the re-entry process in case of future hospitalizations.<br><br>Identify and report strengths and challenges to school and medical team.<br><br>Continue to monitor need for resources available<br><br>The student should be assessed using the Quality of Life measur (or other validated measure) at 6 months from re-entry..	
**Coordinator/point person**	Identify a case coordinator/point person that possesses knowledge across health and education.<br><br>The coordinator/point person will follow the child through the phases.		Identify and set up an home school liaison officer to support the family and pupil in the re-entry process.		A point person should be assigned to check in with the student periodically to assess needs.	The student's re-entry liaison should be checking the re-entry process with the student, parents and teachers.<br><br>A point person should be assigned to check in with the student periodically to assess their needs.	The student checks in with the assigned point person.<br><br>The case coordinator monitors and stays with student, family and school to assess and provide support for successful re-entry.	*To be done at the consensus conference*
**Multidisciplinary care team**	Create a multidisciplinary team in connection with school/services.<br><br>Identify key stakeholders that should take part in the team.		Connect with school/services to determine care team.					*To be done at the consensus conference*

This document contains supplementary material for the above-mentioned article, as provided by the authors.

The original article can be downloaded from https://doi.org/10.5334/cie.159

Phases / Actions	1st hospital Admission	1st hospital stay	1st hospital discharge	Homebound Education	Immediately prior to reentry	During reentry	Following reentry	
	Multidisciplinary team will perform a preliminary meeting in which to discuss the medical case and its assessment.							
**Explain disease and its management in school**	Professionals should educate parents and child on the disease.<br><br>Explain the length of time the student may be in the hospital.<br><br>Educate the students from the main school on classmate disease.<br><br>Educate mainstream school teachers on the pupil's disease.	Explain the nature, characteristics, and consequences (including possible special needs, hospital stay length) of the pupil's disease to parents/carers, teachers, peers, schoolmates and any other relevant stakeholders. It also refers to the parents or the pupil explaining the disease to other stakeholders involved.<br><br>Provide updates to mainstream teachers on the pupil's condition.<br><br>Give the mainstream school teachers an estimate of when the pupil will be returning to school.	Identify new needs the student might have in regards to schooling.<br><br>Explain to family and pupil the updated health condition and how to manage it in regards to school reintegration.<br><br>Provide informative material to the mainstream school in order to support them in educating the classmates on the disease affecting the pupil.<br><br>Create training sessions aimed at mainstream school staff to help them understand what is happening to the diseased pupil.	Educate classmates by providing informative material to the mainstream school in order to support them in educating them on the disease affecting the pupil.<br><br>Educate teachers: create training sessions aimed at school staff to help them understand what is happening to the hospitalized pupil and how to accommodate for the new arisen needs.	The family explains to the mainstream school the pupil's disease and overall situation.<br><br>The hospital staff explains to the mainstream school the pupil's disease and what it entails, including diagnosis, treatment, potential impact on school functioning.<br><br>Send videos explaining the medical characteristics of the disease affecting the pupil and how the treatment works.<br><br>Have a formal meeting to ensure all parties are educated on the pupil's condition and needs.<br><br>Provide education on academic/educational needs, social emotional adjustment and physical medical considerations to parents, pupil, mainstream school staff and classmates.	A doctor and a teacher from the hospital should meet with the class to explain the illness of the pupil.<br><br>Educate peers and teachers on pupil's disease as desired by patient and family.<br><br>Teach to the mainstream school teachers the illness signs and proper ways to react.<br><br>Provide education on academic/educational needs, social emotional adjustment and physical medical considerations to parents, pupil, mainstream school staff and classmates. Offer outreach program.	The mainstream school should be well informed on the pupil's situation.<br><br>Be knowledgeable about the pupil's medical care so to keep him/her healthy and safe.	Written or videotaped informative material.<br><br>… *To be continued at the consensus conference*
**Develop re-entry plan**		Develop the individualized educational plan including educational, social and cultural aspects.	The planning for school re-entry should begin analysing various factors (health, peers, education on disease)		The planning for school re-entry should begin analysing various factors (health, peers, education on disease).<br><br>Pre-plan with school administration the needed accommodations for the pupil.<br><br>Create a specific transition plan for the pupil that includes academic, social/relational and emotional planning.<br><br>The parents and teachers should prepare the pupil to go back to school.		After a year or two the pupil is included into a survivors plan for follow up.	*To be done at the consensus conference*

This document contains supplementary material for the above-mentioned article, as provided by the authors.

The original article can be downloaded from https://doi.org/10.5334/cie.159

Phases / Actions	1st hospital Admission	1st hospital stay	1st hospital discharge	Homebound Education	Immediately prior to reentry	During reentry	Following reentry	
					Liaison between the medical team and the mainstream school around any physical adaptations to facilitate attendance.<br><br>The mainstream school principal should ensure the availability of the necessary human, physical and material resources needed to support the school re-entry.<br><br>The social workers will collaborate with pupil and hospital clinical team in completing a safety plan.			
**Psychological support**	Appointment with clinical psychologist to carry out an evaluation of emotional/psychological/psychosocial status of the pupil and other family members.<br><br>Use validated measures for evaluation.	Provide psychological/psychosocial support to help the pupil process the experience and the trauma.<br><br>Involve play therapists when needed in order to improve child readiness to learn.	Provide psychological/psychosocial support.	Provide psychological/psychosocial support.	Arrange for psychological/psychoeducational assessment and cognitive screening.<br><br>The parents and pupil meet with the department's clinical psychologist for assessment of current pupil's abilities and for counselling.	Provide psychological support within the school in synergy with hospital psychological service.	Provide psychological/psychoeducational/psychosocial assessment.<br><br>Provide psychological support in synergy with the hospital psychological service.<br><br>Provide ongoing psychological/psychosocial support to child and family as needed.	*To be done at the consensus conference*
**Hold meetings**	Perform an initial meeting between staff and carers. When applicable this refers to multidisciplinary staff/carers meetings.<br><br>Prepare parents to carry out a key role in communication, advocacy and liaison with teachers, school and hospital staff.	Prepare parents to carry out a key role in communication, advocacy and liaison with teachers, school and hospital staff.<br><br>Hold meetings between staff/multidisciplinary staff-carers meeting.<br><br>Hold meetings taking into great consideration the student point of view.	Schedule a transition meeting with multidisciplinary team and other stakeholders involved.<br><br>Schedule a supportive re-entry meeting.	Create meeting opportunities in the presence of the school teachers.	Have a formal meeting with teachers and members of the medical team to ensure all parties are educated on the pupil's condition and needs.<br><br>Set up a formal re-entry meeting with stakeholders, family and pupil.	A doctor and a teacher from the hospital should organize a cultural meeting with the class and other relevant stakeholders.<br><br>Set up a formal re-entry meeting with stakeholders, family and pupil. This meeting should be carried out with supportive and restorative energy.	Organize live or virtual meetings between medical staff, hospital teacher and school staff.	*To be done at the consensus conference*

This document contains supplementary material for the above-mentioned article, as provided by the authors.

The original article can be downloaded from https://doi.org/10.5334/cie.159
