# Supplementary material for: Pathways to School Reentry for Children and Young People with a Medical or Mental Health Condition: An International Delphi Study
Source: Contin Educ. 2025 Mar 5;6(1):38–57. doi: 10.5334/cie.159 (PMC11887473; doi:10.5334/cie.159)
Supplement: Supplementary File 3. — Poster with the Physical H-SRM. [file cie-6-1-159-s3.pdf]

School reentry model for learners with a physical health condition

Please note that the following interventions are presented as general suggestions and guidelines.
They must always be tailored to the specific diagnosis and condition of the learner, their family, and life context.
It is important to recognize that not all suggested activities may be appropriate or beneficial for every individual. If there is any doubt, it is advisable to check ideas at a multidisciplinary level.

Glossary and Language Description:

The term **learner** is used to encompass primary and secondary school levels.
Mainstream school refers to the school that the learner typically attends.
Home schooling refers to the situation where a learner is taught at home due to illness preventing them from attending their mainstream school. In this case, they may receive home visits from a teacher or engage in online teaching.

TIMEFRAME

BEFORE REENTRY | **REENTRY** | **POST REENTRY** | **TOOLS AND ACTIVITIES**

ACTION

- **Before reentry – 1st HOSPITAL ADMISSION:** The learner is admitted to the hospital for the first time
- **Before reentry – 1st HOSPITAL STAY:** The learner stays in the hospital for treatment
- **Before reentry – 1st HOSPITAL DISCHARGE:** The learner is discharged from the hospital
- **Before reentry – HOME SCHOOLING:** The learner stays at home where they receive their education
- **Reentry – IMMEDIATELY BEFORE REENTRY:** The learner is about to return to school.
- **Reentry – DURING REENTRY:** The learner returns to their original school
- **Post reentry – FOLLOWING REENTRY:** The learner is back to attending the mainstream school

1. WELCOMING

Facilitate a flexible, friendly and supportive environment.

1st Hospital admission: Prepare the learner and family for hospitalization.
Allow parents and learners to voice any questions or concerns regarding the hospital school.

Introduce teachers and key ward staff to patients and caregivers and explain their roles in the department.
Be mindful not to overwhelm learners and families with excessive information or to provide it at an inappropriate time.

1st Hospital stay: Introduce the hospital school to learners and parents, inspiring and motivating them to continue their education.

See tools: You can use synchronous or asynchronous communication tools, depending on the patient's condition.

Tools and activities:
Use virtual guides and tours to address questions, fears, and concerns before admission.
Provide a ward map.
Display a "who-is-who" billboard with staff pictures and roles.
Create an "identity card" or "all about me" profile for the learner to share their interests and goals.
Give each new learner a welcome postcard with contacts, a QR code to the school webpage, and post box locations for messages.

2. INFORMED CONSENT

Informed consent should be viewed as an ongoing process, not as a single, static event.
Approach the child and family with kindness and respect, adapting communication to be culturally sensitive and inclusive.
The learners with a chronic illness should actively participate in the entire process and articulate their perspectives to ensure their voices are heard, considering their continuously evolving abilities.
Professionals should explain the pros and cons of disclosure to the learners and their families and inform them of their rights/responsibilities and effective approaches, helping them to decide what information to share, when, and with whom.

1st Hospital admission: Before commencing hospital education, obtain consent from parents/carers, and any other relevant stakeholders.
Inform the relevant parties that their information can be shared with different counterparts at different stages, reflecting the distinct needs and circumstances of everyone involved.
Underaged learners can express their assent in verbal form.

1st Hospital stay: Have a parent/guardian sign a consent form to contact the mainstream school/key stakeholders outside of the hospital system.

Home schooling: Obtain consensus between the health and educational teams, the family, and the children on the modalities and content of information that will be exchanged with the mainstream school.
If teachers intend to arrange a conference call involving the learner's classroom, privacy consent from parents in the mainstream school is required.

Immediately before reentry: Staff should also be consulted before meeting with the learner about any questions and suggestions on how to approach disclosure at this stage.
Talk to the learner about how to describe their illness/condition to their peers or how to redirect the conversation if they don't want to talk about it.
Educate the learner on how to express their preference of "I do not wish to discuss this" if they so desire.

During reentry: Support can be provided for the learner, peers/staff, and parents by offering tools and practical methods for disclosing information.
This includes guidance on how to use and share the information, understanding the practical implications of the medical condition, and promoting multidisciplinary collaboration among all parties involved.

Following reentry: Communicate the potential impact of disclosure to the learner and their family and provide the appropriate support to help them effectively cope with any challenges that may arise.

Tools and activities:
Recording tools and case story.
Peer interview recordings.
Class project with Powtoons.
Short presentation about consent.

3. EMPOWER COMMUNICATION AND CONNECTIONS

Create and maintain a communication link between the family, hospital school, and mainstream school to create a shared work plan, minimize social isolation and school difficulties, and keep everyone informed of the learner's academic progress.
Create and maintain connections between the learner with a medical condition, their classmates, and teachers, to promote social participation and consistently empower their sense of belonging. These may include hospital/home visits from classmates and mainstream schoolteachers.

1st Hospital admission: **See *Action 2. Informed Consent.***

Contact the learner's mainstream school to inform them that you have taken the learner on roll and start gathering academic data.
Initiate contact promptly to exchange ideas and information, focusing on collaboration rather than immediate learning.

An effective electronic system is essential to store all the information, meeting notes, and actions from meetings.
Additionally, formal written reports to the mainstream school are crucial for communication and to support the learner.

1st Hospital stay: The hospital school can liaise with the mainstream school to advocate and support the learner to remain engaged with the social and emotional aspects of their development alongside their education. If deemed possible, identify one or more classmates to keep the learner updated on their mainstream school life.
Mainstream and hospital schools should communicate regularly to develop joint activities that align with both institutions.
Distant learning activities should be organized (synchronously or asynchronously) with the mainstream class and classmates.

Provide regular progress reports to the mainstream school if the hospital duration is longer than two weeks.

1st Hospital discharge to during reentry: **The hospital teacher is expected to be available during various stages to maintain communication with the learner and provide support to the home- and mainstream school teachers. This exchange is crucial for understanding learner behavior and tailoring strategies and educational resources to meet the specific needs of each learner.**

1st Hospital discharge: Provide all the relevant information collected during their stay in the hospital school to the mainstream school teachers to make them part of the educational team.

Home schooling: Regular communication between the homeschool and mainstream teachers is essential to ensure that the teaching content and modality are tailored to the l earner's educational requirements and are aligned with the class.

Learning at home could include online learning options and the possibility to connect with the learner's own classroom, schoolmates, and/or teachers, where appropriate.

Immediately before reentry: **See *Action 6. Coordinator.***

Perform a peer presentation when needed, and if appropriate to the learner with a medical condition.

During reentry: It is important to foster inclusive peer groups that understand and embrace diversity, thereby mitigating and discouraging bullying related to their illness.

Create a sense of normality in the learner's life to make it more similar to that of their peers.

Tools and activities:
Telephone and conference calls, and any relevant technology that can be used to maintain contact.
Specific outreach and connecting programs such as:
Seesaw (https://www.seesaw.org.uk)

Monkey In My Chair (http://www.monkeyinmychair.org)

The use of hybrid virtual classrooms to help learners to reenter or even start in a new school or class.

4.ASSESS, MONITOR, AND SUPPORT

Conduct an initial assessment and continue to monitor the learner's academic and social-emotional needs, strengths, and challenges.

1st Hospital admission: Before the learner begins formal schooling at the hospital, assess their psychological and educational readiness to determine if they can cope with the demands of school.

In situations where resources are limited, conducting a multi-tiered risk assessment of the student and the family's socio-ecological status can help in calibrating the necessary level of interventions where they are most critical.

1st Hospital stay: Provide support to the learner's family/parents/siblings as needed.

Wherever possible, consider assessing family and learner needs that may impact the child's education and wellbeing, promoting positive functioning and academic growth, and providing strategies for positive family coping strategies (e.g., the importance of sleep, coping skills, and social support).

1st Hospital discharge: The hospital teacher prepares a report detailing completed work and identifying areas where the learner needs continued support at home.

Identify the needs for home schooling, technical, and educational support and provide adequate help.

Home schooling: Support the mainstream school to organize and participate in the home lessons.

Immediately before reentry: Assess learner's and family's readiness for school reentry and possibile needs for support.

The school principal should evaluate the extent to which the mainstream school is prepared to support the student's reentry and provide the necessary adaptations and resources.

Organize meetings with the family, mainstream school teachers, clinicians, and liaison teachers to discuss support strategies and ongoing needs such as an individualized learning plan (ILP), a risk management plan (RMP), modified and flexible timetables, etc.

During reentry: Verify that the accommodations/modifications are put in place.

Frequently check in with the learner, family, and teachers to discuss the reintegration process and support provided, and ensure that the accommodations are being applied and that all learners are safe and satisfied with them.

If the learner is experiencing academic challenges refer them for an assessment.

A psychologist monitors the learner's reentry process and liaises with the school and parents.

Following reentry: Monitor the learner for bullying due to their illness.

Have a planned follow-up on the transition plan with the learner and mainstream school staff.
Continuously monitor the learner's attendance and address any issues that may have occurred at school.
Regularly evaluate and modify support for the learner and their family based on their reentry experience.

Identify and report strengths and challenges to the school and medical team.

Identify successful practices for handling the reentry process in case of future hospitalizations.

Tools and activities:
Pair the learner with a chosen supportive peer for social, emotional, and academic help during reintegration. Rotate buddies to avoid overburdening one peer.
Encourage the learner to keep a journal of their feelings, challenges, and achievements. Family and professionals can also use this approach.
Use visual tools to help younger learners express their daily feelings and specific concerns (e.g., peers, academics, energy levels).
Provide a short questionnaire for learners, parents, medical team, and teachers to complete. For example: www.sdqinfo.org.

5.TAILORED LESSON PLANNING, DELIVERY, AND REPORTS

Do not compare learners' academic abilities to others; explain to everyone that learning is individualized.
The individualized learning plan is adapted over time according to the child's health status and should prioritize a workload that is deemed essential for the learner's academic or socio-emotional progress.
To prevent overload and school fear, adjust the daily amount of schooling with a light timetable, flexible learning materials, and playful activities.

1st Hospital admission to 1st hospital stay: **Gather information from the learner, parents, and mainstream school about their needs, priorities, concerns, and strengths related to the learner's academic performance and overall wellbeing.**

1st Hospital discharge to following reentry: **Check if the learner needs specific academic support or other compensatory services.**
Promote individualized education and special attention among all the teachers to guarantee equity.

1st Hospital admission: Hospital and mainstream teachers cooperate to create a plan for schoolwork based on the information collected.
Education goals are determined and shared between the learner and all the stakeholders.

Discuss the parents' rights and school responsibilities in serving learners with special needs/health conditions.

1st Hospital stay: Once stressors and risk factors are identified, identify strategies and resources to provide targeted support to the learner.

Explore and implement flexible alternative teaching methods and accommodations, while aligning with the mainstream curriculum.

Offer special exam regulations when necessary (e.g., extra time, fewer questions).

1st Hospital discharge: The hospital school teachers should identify learning material in conjunction with mainstream school teachers, provide the learners with school supplies and activities, and encourage them to do the schoolwork at home.

Home schooling: Home tuition can be organized by the mainstream school and carried out by different figures (e.g., a Home Liaison Officer).

A hospital school report should be given to the teacher for the home tuition of the learner.

Some distant learning activities can also be done with mainstream teachers and the learner's original class.

Immediately before reentry: Create a transition plan covering academic, social, and emotional aspects to facilitate attendance.
Support the learner with shorter days or revised timetables as needed.
Provide the learner with a space to discuss expectations, feelings, or worries about reentry. Foster hope and optimism.
Ensure the learner can safely participate in activities involving sports, play, or physical effort (e.g., arts, PE, outdoor activities).

During reentry: Educational professionals should be aware and flexible regarding what is happening, and the adjustments needed.

Create a plan with schools and teachers in case of future hospitalizations.

Following reentry: Promote independence and growth for the child as appropriate.

After a couple of years, the learner is enrolled in a follow-up group.

Tools and activities:
Play-based learning activities are recommended for engagement.

Utilize self-assessment tools to engage the learner in the planning process, allowing them to express their interests and set attainable academic and social personal goals.

Consider using a badge or point system.

Offer the use of computers instead of handwriting when applicable.

6. COORDINATOR

There should be a case coordinator for health.
There should be an educational care coordinator who acts as project manager and liaison, taking care of networking and helping families with their child's education.

1st Hospital admission: The coordinators attend all the meetings and communicate with the school, learners, parents, and medical practitioners.

1st Hospital stay: The educational care coordinator manages communication with the schools and ensures all relevant consents are given.

See Action 2. Informed Consent, and Action 3. Empower Communication, and Connections.

1st Hospital discharge: The educational care coordinator supports the family and learner in the reentry process.

Contact possible community services that can provide education support and leisure activities for the child during the next phases.

Home schooling: Supervise the organization of school at home and other education and/or leisure activities for the learner.

Identify specialists for specific learners' needs related to issues/difficulties during the initiation of home-based education.

Immediately before reentry: ***See Actions 2. Informed Consent and 4. Assess, Monitor, and Support.***

The educational care coordinator informs the mainstream school about the learner's discharge and provides relevant academic and social information.
The healthcare coordinator provides diagnostic information about the learner's health and advises on necessary precautions in the mainstream school.

During reentry: ***See Action 4. Assess, Monitor and Support.***

Following reentry: ***See Action 4. Assess, Monitor and Support.***

7. MULTIDISCIPLINARY CARE TEAM

A multidisciplinary team follows the entire education process in connection with the school and other services. The team's composition varies depending on the specificities of the illness and context.

1st Hospital admission: Identify the key stakeholders and hold an initial meeting between staff and carers.

The learner should be considered "part" of the multidisciplinary team. This will vary with age as appropriate.

1st Hospital stay: Teachers work closely with other hospital specialists and therapists to implement strategies that cater for the learner's diverse needs, including speech-language, occupational, and physical therapy.

1st Hospital discharge to following reentry: **The hospital, home–school teachers, and education liaison work together to assist the learner's mainstream school to support the learner academically and socially.**

1st Hospital discharge: The multidisciplinary team will meet to discuss the medical case, its assessment, and provide support in a final meeting before discharge.

Immediately before reentry: Hold a formal meeting to ensure all parties are educated on the learner's condition and needs at this stage.

8. EXPLAIN THE ILLNESS AND ITS MANAGEMENT IN SCHOOL

See Action 2 (Informed Consent) before initiating any activity in this section!

Healthcare professionals have a responsibility to provide comprehensive and context-appropriate disease information to all stakeholders, including the young patient and their peers.
Education professionals are responsible for facilitating the delivery of appropriate educational, socio-emotional, and medical information to the school staff and schoolmates.

1st Hospital admission: Explain how long the learner may need to stay in the hospital, or why it is not possible to determine this information in advance.

Inform classmates and teachers about the learner's hospitalization.

1st Hospital stay: Provide updates to mainstream teachers on the learner's condition.

When possible, give the mainstream school teachers an estimate of when the learner will be returning to school.

1st Hospital discharge to following reentry: **Provide informative materials and/or training to the mainstream school staff and classmates to support them in understanding what is happening to the learner and how to accommodate their new needs.**

Immediately before reentry: ***See Action 4. Assess, Monitor, and Support.***

Provide training for the school community and schoolmates to help them understand their classmate's short and long-term challenges connected to the illness and its management in school (educational, social, physical education...).

During reentry: A doctor and a teacher from the hospital should meet with the class and other relevant stakeholders to explain the learner's illness.

Explain the signs of illness and the correct ways to react to the learner's mainstream school teachers.

Offer an outreach program.

Following reentry: Schools should remain knowledgeable about the learner's medical care to keep him/her healthy and safe.

Tools and activities:
Written or recorded information.

Podcast with doctors, teachers, and parents about illness experiences

Support the learner to use the Pediatric Brain Tumor Foundation's Imaginary Friends Society clips to talk to their peers: www.imaginaryfriendsociety.com/home.

Role-playing where the learner anticipates explaining why they were in the hospital for so long.

9. PSYCHO EDUCATIONAL SUPPORT

Provide psychological and/or psychosocial support to the child, family, and siblings as needed during the different phases of the illness pathway.

1st Hospital admission: Appointment of a liaison person to evaluate the learner's emotional, psychological, psychosocial status and that of other family members.

See comments on multi-tiered system at ***4. Assess, Monitor, and Support.***

1st Hospital stay: Based on the illness and therapies, it may be useful to involve specific therapists when needed to improve the child's readiness to learn.

Immediately before reentry: Arrange for assessment and cognitive screening if the illness and therapies indicate it is necessary.
Involve specific therapists when needed to improve child readiness to learn.

Capurso, Moracci, Borsci

Pathways to school reentry for children and young people with a medical or mental health condition: an international Delphi study

Supplementary Material 3

A copy of this resource can be downloaded at https://doi.org/10.5334/cie.159
