# Supplementary material for: Pathways to School Reentry for Children and Young People with a Medical or Mental Health Condition: An International Delphi Study
Source: Contin Educ. 2025 Mar 5;6(1):38–57. doi: 10.5334/cie.159 (PMC11887473; doi:10.5334/cie.159)
Supplement: Supplementary File 4. — Poster with the Mental H-SRM. [file cie-6-1-159-s4.pdf]

School reentry model for learners with a mental health condition

Please note that the following interventions are presented as general suggestions and guidelines.
They must always be tailored to the specific mental health condition of the learner, as well as their family and life context.
It is important to recognize that not all suggested activities may be appropriate or beneficial for every individual. Whenever in doubt, it is advisable to check ideas with the mental health team.

Glossary and Language Description:
The term learner is used to encompass both primary and secondary school levels.
Mainstream school refers to the school that the learner typically attends.
Home schooling refers to the situation where a learner is taught at home due to illness preventing them from attending their mainstream school. In this case, they may receive home visits from a teacher or engage in online teaching.
Note: "Home education" has been removed as a specific time phase. This should only be a last resort for patients with mental health needs as it can lead to social isolation and disengagement.

T I M E F R A M E

ACTION	BEFORE REENTRY			REENTRY	POST REENTRY	TOOLS AND ACTIVITIES
	1ST HOSPITAL ADMISSION<br>The learner is admitted to the hospital for the first time	1ST HOSPITAL STAY<br>The learner stays in the hospital for treatment	1ST HOSPITAL DISCHARGE & IMMEDIATELY BEFORE REENTRY<br>The learner is discharged from the hospital and is about to return to school	DURING REENTRY<br>The learner returns to their original school	FOLLOWING REENTRY<br>The learner is back to attending the mainstream school	

1. WELCOMING

Facilitate a flexible, confidential, friendly, and supportive environment. (1st Hospital Admission – 1st Hospital Stay)

1st Hospital Admission:
Prepare the child and family for hospitalization.
Allow parents and learners to voice any questions or concerns regarding the hospital school.
Introduce teachers and key ward staff to patients and caregivers, and explain their roles in the department.
Be mindful not to overwhelm learners and families with excessive information or to provide it at an inappropriate time.

1st Hospital Stay:
Introduce the learners and parents to the hospital school and inspire and motivate them to continue with their education.

See tools: you can use synchronous or asynchronous communication tools, depending on the type of hospitalization.

Tools and Activities:
Use virtual guides and tours to address questions, fears, and concerns before admission.
Provide a ward map.
Display a "who-is-who" billboard with staff pictures and roles.
Create an "identity card" or "all about me" profile for the learner to share their interests and goals.

Give each new learner a welcome postcard with contacts, a QR code to the school webpage, and postbox locations for messages.

2. INFORMED CONSENT

The principle of allowing learners with mental health needs to actively participate in decision-making activities should always be tailored to the individual's condition, as this can cause excessive stress.
Informed consent should be viewed as an ongoing process, not as a single, static event.
Ensure that the child and family are approached with the utmost kindness and respect. Effectively adapt communication to respect and accommodate diverse cultures by using culturally sensitive and appropriate forms of communication.
Professionals should explain the pros and cons of disclosure to the learners and their family, and inform them of their rights/responsibilities and effective approaches, helping them to decide what information to share, when, and with whom.

1st Hospital Admission:
Before commencing hospital education, obtain consent from parents/carers, and any other relevant stakeholders.
Inform the relevant parties that their information can be shared with different counterparts at different stages, reflecting the distinct needs and circumstances of everyone involved.
Underaged learners can express their assent in verbal form.

1st Hospital Stay:
Have the parent/guardian sign a consent form to contact the mainstream school/key stakeholders outside of the hospital system.

1st Hospital Discharge & Immediately Before Reentry:
Staff should also be consulted before meeting with the learner about any questions and suggestions on how to approach disclosure at this stage.
Talk with the learner about how to describe their illness/condition to their peers or how to redirect the conversation if they don't want to talk about it.
Educate the learner how to express their preference of "I do not wish to discuss this" if they so desire.

During Reentry:
Support can be provided for the learner, peers/staff, and parents by offering tools and practical methods for disclosing information. This includes guidance on how to use and share the information, understanding the practical implications of the condition, and promoting multidisciplinary collaboration among all parties involved.

Following Reentry:
Communicate the potential impact of disclosure to the learner and their family and provide the appropriate support to help them effectively cope with any challenges that may arise.

Tools and Activities:
Video tools and case story.
Peer interview.
Class project with Powtoons.

Up To Me is a valuable tool that helps students reflect and decide whether and how they would like to disclose.
https://eliminatestigma.org/up-to-me/what-is-up-to-me/

3. EMPOWER COMMUNICATION AND CONNECTIONS

A practitioner should be consulted before any activities that involve a connection between a learner with mental health needs and their peers.
Create and maintain a communication link between the family, hospital school, and mainstream school to create a shared work plan, to minimize social isolation and school difficulties, and keep everyone informed of the learner's academic progress.
Whenever possible, create and maintain connections between the learner with mental health needs, their classmates, and teachers, to promote social participation and constantly empower the sense of belonging. Such communication with the school should also involve a degree of preparation both of the learner and their peers.

1st Hospital Admission:
See *Action 2. Informed Consent.*

Contact the child's mainstream school to inform them that you have taken the learner on roll and start gathering academic data.
Initiate contact promptly to exchange ideas and information, focusing on collaboration rather than immediate learning.
An effective electronic system is essential to store all the information, meeting notes, and actions from meetings.
Additionally, formal written reports to the mainstream school are crucial for communication and to support the child.

1st Hospital Stay:
The hospital school can liaise with the mainstream school to advocate and support the learner to remain engaged with the social and emotional aspects of their development alongside their education.
If deemed possible and appropriate, select one or more classmates to take turns keeping the learner updated on their mainstream school life. However, bear in mind that in some cases, the school itself could be perceived as part of the problem. Additionally, the proper time to initiate this connection should be agreed upon with the medical team.
Mainstream and hospital schools should communicate regularly to create (collaborative) activities that align with both institutions.
Provide regular progress reports to the mainstream school if the hospital duration is longer than two weeks.
Appropriate forms of communication should also be encouraged within the hospital school between learners.

The hospital teacher is expected to be available during various stages to maintain communication with the learner and provide support to the home and mainstream school teachers. This exchange is crucial for understanding learner behavior and tailoring strategies and educational resources to meet the specific needs of each learner. (1st Hospital Discharge – During Reentry)

1st Hospital Discharge & Immediately Before Reentry:
See *Action 6. Coordinator.*

Provide all the relevant information collected during their stay in the hospital school to the mainstream school teachers to make them part of the educational team.

Following Reentry:
It is important to foster inclusive peer groups that understand and embrace diversity, thereby mitigating and discouraging bullying related to the learner's illness.
Create a sense of normalcy in the learner's life so that it more closely aligns with the experiences of their peers.

Tools and Activities:
Telephone, conference calls, and any relevant technology that can be used to maintain contact.
Specific outreach and connecting programs such as:
Seesaw (https://www.seesaw.org.uk),
Monkey In My Chair (http://www.monkeyinmychair.org).

You may use hybrid virtual classrooms to help learners to reenter or even start in a new school or class.

Check more resources at.
https://eliminatestigma.org.

4. ASSESS, MONITOR, AND SUPPORT

See *Action 5. Tailored lesson planning, delivery, and reports.*

Perform an initial assessment and continue to monitor the learner's academic and social-emotional needs, strengths, and challenges. Such an assessment should involve listening to the learner's voice and gradually making them self-aware of- and protagonists in their academic and social functioning processes.

1st Hospital Admission:
Before the learner begins formal schooling at the hospital, assess their psychological and educational readiness.
Recognize that hospital schooling is a multidisciplinary decision integral to recovery and should start as early as possible.

1st Hospital Stay:
Provide support to the learner's family/parents/siblings as needed.

Wherever possible, consider also assessing family and learner needs that may impact the child's education and well-being, promoting positive functioning and academic growth, and providing strategies for positive family coping (e.g., importance of sleep, coping skills, social support).

1st Hospital Discharge & Immediately Before Reentry:
The hospital teacher produces a report that is shared with the stakeholders and states the work done and where the learner should obtain support once they are back home.

The school principal should evaluate the extent to which the mainstream school is prepared to support the student's reentry and provide the necessary adaptations and resources.

The medical team and hospital teachers should assess the readiness of the child and family to be reintegrated into the mainstream school environment.

See *Action 8. Explain the Illness and its Management in school*

Monitor the learner for bullying due to their illness. (During Reentry – Following Reentry)

During Reentry:
Verify that the accommodations/modifications are put in place.
Frequently check in with the learner, class, family, and teachers to discuss the reintegration process, support provided, and ensure that the accommodations are being applied and the learner is satisfied with them.
If the learner is experiencing academic challenges, refer them for an assessment by an appropriate professional.
The buddies and other classmates may need some type of support too. Ensure professional resources are available for the whole class.

Following Reentry:
Have a planned follow-up on the transition plan with the learner and mainstream school staff.
Continuously monitor the learner's attendance and address any issues that may have occurred at school.
Regularly evaluate and modify support for the learner and their family based on their reentry experience.
Identify and report strengths and challenges to the school and medical team. Identify those practices that were successful in handling the reentry process in case of future hospitalizations.

Tools and Activities:
Buddy system at a peer level.
A personal coach (adult or older peer) for specific activities or problemS. The learner could indicate them, but rotate buddies to avoid overburdening one peer.
Use visual tools to help learners express their daily feelings and specific concerns (e.g., with peers, academics, energy levels).
Tools: "problem-based contacts" for the learner, classroom teacher, principal, and parents.
E.g., for a learner with anxiety disorder this could be:
1) Use skills learned at the hospital;
2) Leave the classroom and go to a quiet room for breathing exercises;
3) Go to the principal's office to ring their parents/therapist.

Keep a journal of feelings, challenges, and achievements - a method also useful for family and professionals.

5. TAILORED LESSON PLANNING, DELIVERY, AND REPORTS

Do not compare learners' academic abilities to others; explain to everyone that learning is individualized.
The individualized learning plan evolves over time and should prioritize a workload that is deemed essential for the learner's academic and socio-emotional progress.
To prevent overload and school fear, adjust the daily amount of schooling with a light timetable, flexible learning materials, and playful activities.
Maintaining a collaborative environment, particularly between the learner and the teacher, is crucial.

Gather information from the learner, parents, and mainstream school about their needs, priorities, concerns, and strengths related to the learner's academic performance and overall wellbeing.
Education and social activities should take place in non-medical spaces. (1st Hospital Admission – 1st Hospital Stay)

See *Action 4. Assess, Monitor and Support*
Check if the learner needs specific academic support or other compensatory services.
Promote individualized education and special attention among all the teachers to guarantee equity. (1st Hospital Discharge – Following Reentry)

1st Hospital Admission:
Hospital and mainstream teachers cooperate to create a plan for schoolwork based on the information collected.
Education goals are determined and shared between the learner and all the stakeholders.
Discuss the parents' rights and school responsibilities in serving learners with special needs/conditions.

1st Hospital Stay:
Once stressors and risk factors are identified, identify strategies and resources to provide targeted support to the learner.
Explore and implement flexible alternative teaching methods and accommodations, while aligning with the mainstream curriculum.
Offer special exam regulations when necessary (e.g., extra time, fewer questions).

1st Hospital Discharge & Immediately Before Reentry:
Create a specific transition plan for the learner that includes academic, social/relational and emotional planning that will facilitate attendance.
The mainstream teacher should offer the learner a place for discussion to express feelings, worries, doubts.

Take time to communicate with the learner about reentry to their mainstream school. Talk about their thoughts, fears, expectations. Talk about practical things. Create hope and optimism.

Ensure the learner can safely participate in activities involving sports, play, or physical effort (e.g., arts, PE, outdoor activities).
Encourage participation to social activities, as social goals are just as important as cognitive goals.

During Reentry:
Educational professionals should be aware and flexible towards what is happening and the adjustments needed.

When needed, the mainstream school could introduce an appropriate number of daily hours/weekdays.

Create a plan with school and teachers in case of future hospitalizations.

Following Reentry:
Promote independence and growth for the learner as appropriate.

Tools and Activities:
Play-based learning activities recommended for engagement.

Utilize self-assessment tools to engage the learner in the planning process, allowing them to express their interests and set attainable academic and social personal goals.

Consider offering the use of computers instead of handwriting when applicable.

6. COORDINATOR

There should be a Mental Health Case Coordinator (or equivalent role), who should serve as the main contact between patients and services, supporting person-centered decisions and streamlining information for clinicians, patients, and families.
There should be an educational care coordinator who acts as project manager and liaison, taking care of networking and helping families with their child's education.

1st Hospital Admission:
The coordinators attend all the meetings and communicates with the school, learner, parents, and medical practitioners.

1st Hospital Stay:
The educational care coordinator manages communication with the schools and ensures all the relevant consents are given.

See *Actions 2. Informed Consent* and *3. Empower Communication and Connections.*

1st Hospital Discharge & Immediately Before Reentry:
The educational care coordinator supports the family and learner with the reentry process.
Contact possible community services that can provide education support and leisure activities for the child during the next phases.
The educational care coordinator informs the mainstream school about the learner's discharge from the hospital and provides them with the relevant academic and social information.
A healthcare case coordinator provides pertinent diagnostic information regarding the learner's health and advises on precautions in the mainstream schools.

See *Actions 2. Informed Consent* and *5. Assess, Monitor and Support.*

During Reentry:
See *Action 5. Assess, Monitor and Support.*

Following Reentry:
See *Action 5. Assess, Monitor and Support.*

7. MULTIDISCIPLINARY CARE TEAM

A multidisciplinary team follows the entire education process in connection with school/services. The team should have professionals from health and education and across settings (e.g., hospital, school, community services) who jointly follow a child and family centered approach.
The child and the family should be considered "part" of the multidisciplinary team. This will vary with age and conditions as appropriate.

1st Hospital Admission:
Identify the key stakeholders and hold an initial meeting between staff and carers.
The child should be considered "part" of the multidisciplinary team. This will vary with age as appropriate.

1st Hospital Stay:
Teachers work closely with other hospital specialists and therapists to implement strategies that cater for the learner's diverse needs, including speech-language, occupational, and physical therapy.

The hospital, home school teachers, and education liaison work together to assist the learner's mainstream school to support the learner academically and socially. (1st Hospital Discharge – Following Reentry)

1st Hospital Discharge & Immediately Before Reentry:
The multidisciplinary team meets to discuss the medical case, its assessment, and provide support in a final meeting before discharge.
Reentry meeting with the hospital school teacher, classroom teacher, principal, parents, and learner to discuss the topics covered at the hospital school, skills learned to manage their mental health needs, parent's expectations, and the learner's needs.
Organize meetings with the family, mainstream school teachers, clinicians, and liaison teacher to discuss support strategies and ongoing needs such as an ILP (individualized learning plan), an RMP (risk management plan), modified and flexible timetables, etc.

8. EXPLAIN THE ILLNESS AND ITS MANAGEMENT IN SCHOOL

See *Action 2. informed Consent* before initiating any activity of this section!
The learner with mental health needs should always have control over deciding what and how to disclose.
Mental health professionals have a responsibility to provide comprehensive and context-appropriate information about the condition to all stakeholders, including the young patient and their peers.

1st Hospital Admission:
Explain how long the learner may need to stay in the hospital, or why it is not possible to determine this information in advance.
Inform classmates and teachers about the learner's hospitalization.

1st Hospital Stay:
Provide updates to mainstream teachers on the learner's condition.
When possible, give the mainstream school teachers an estimate of when the learner will be returning to school.

Provide informative materials and/or training to mainstream school staff and classmates to help them understand the learner's situation and effectively accommodate any newly arising needs. (1st Hospital Discharge – Following Reentry)

1st Hospital Discharge & Immediately Before Reentry:
See Action *4. Assess, Monitor and Support.*

Consider activities or advice for the student's classmates who play a central role in the reception of their schoolmate.

During Reentry:
Multidisciplinary team representatives should meet with the class and relevant stakeholders to explain the learner's mental health condition, including behavioral signs and appropriate responses for mainstream teachers and school personnel.
Recognize the importance of peer support by discussing supportive strategies with both the class and the learner when appropriate.
Address the potential issue of bullying and involve the class in activities designed to foster a protective environment.
Offer an outreach program.

Tools and Activities:
Provide written or recorded information.

Podcast with learners, doctors, teachers, and parents about illness experiences.

Role-playing where the learner anticipates explaining why they were in the hospital for a long time.

Check resources at
https://eliminatestigma.org

9. PSYCHO EDUCATIONAL SUPPORT

Provide psychological and/or psychosocial support to the child, family, and siblings as needed during the different phases of the illness pathway.
Consider providing support to the class as well.

1st Hospital Admission:
Appointment with a psychologist to conduct an evaluation of the learner's emotional/psychological/psychosocial status and other family members.

A multi-tiered risk assessment of the student and the family's socio-ecological status can help in calibrating the necessary level of interventions where they are most critical.

1st Hospital Stay:
Based on the condition and therapies, it may be useful to involve specific therapists when needed to improve the child's readiness to learn.

Outpatient therapeutic services or community centers may provide support with specific mental health, social or recreational programs. (1st Hospital Discharge – Following Reentry)

1st Hospital Discharge & Immediately Before Reentry:
If the condition and therapies indicate it may be necessary, arrange psychosocial assessment and cognitive screening.

During Reentry:
A psychologist follows the learner's reentry process and cooperates with the school and parents.

Capurso, Moracci, Borsci

Pathways to school reentry for children and young people with a medical or mental health condition: an international Delphi study

Supplementary Material 4

A copy of this resource can be downloaded at https://doi.org/10.5334/cie.159
